# Breast cancer prevention by short-term inhibition of TGFβ signaling

**DOI:** 10.1038/s41467-022-35043-5

**Published:** 2022-12-07

**Authors:** Maša Alečković, Simona Cristea, Carlos R. Gil Del Alcazar, Pengze Yan, Lina Ding, Ethan D. Krop, Nicholas W. Harper, Ernesto Rojas Jimenez, Donghao Lu, Anushree C. Gulvady, Pierre Foidart, Marco Seehawer, Benedetto Diciaccio, Katherine C. Murphy, Jason Pyrdol, Jayati Anand, Kodie Garza, Kai W. Wucherpfennig, Rulla M. Tamimi, Franziska Michor, Kornelia Polyak

**Affiliations:** 1 Department of Medical Oncology, Dana-Farber Cancer Institute, Boston, MA 02215 USA; 2 Department of Medicine, Brigham and Women’s Hospital, Boston, MA 02115 USA; 3 Department of Medicine, Harvard Medical School, Boston, MA 02115 USA; 4 Department of Data Science, Dana-Farber Cancer Institute, Boston, MA 02215 USA; 5 Department of Stem Cell and Regenerative Biology, Harvard University, Cambridge, MA 02138 USA; 6 Department of Biostatistics, Harvard T. H. Chan School of Public Health, Boston, MA 02115 USA; 7 Channing Division of Network Medicine, Brigham and Women’s Hospital, Boston, MA 02115 USA; 8 Department of Epidemiology, Harvard T. H. Chan School of Public Health, Boston, MA 02115 USA; 9 Department of Cancer Immunology and Virology, Dana-Farber Cancer Institute, Boston, MA 02215 USA; 10 Department of Immunology, Harvard Medical School, Boston, MA 02115 USA; 11 Center for Cancer Evolution, Dana-Farber Cancer Institute, Boston, MA USA; 12 The Broad Institute of MIT and Harvard, Cambridge, MA 02138 USA; 13 The Ludwig Center at Harvard, Boston, MA 02115 USA; 14 Harvard Stem Cell Institute, Cambridge, MA 02138 USA

**Keywords:** Breast cancer, Mammary stem cells, Cancer prevention

## Abstract

Cancer prevention has a profound impact on cancer-associated mortality and morbidity. We previously identified TGFβ signaling as a candidate regulator of mammary epithelial cells associated with breast cancer risk. Here, we show that short-term TGFBR inhibitor (TGFBRi) treatment of peripubertal ACI inbred and Sprague Dawley outbred rats induces lasting changes and prevents estrogen- and carcinogen-induced mammary tumors, respectively. We identify TGFBRi-responsive cell populations by single cell RNA-sequencing, including a unique epithelial subpopulation designated secretory basal cells (SBCs) with progenitor features. We detect SBCs in normal human breast tissues and find them to be associated with breast cancer risk. Interactome analysis identifies SBCs as the most interactive cell population and the main source of insulin-IGF signaling. Accordingly, inhibition of TGFBR and IGF1R decrease proliferation of organoid cultures. Our results reveal a critical role for TGFβ in regulating mammary epithelial cells relevant to breast cancer and serve as a proof-of-principle cancer prevention strategy.

## Abstract (web summary)

TGFβ signalling is reported to regulate hormone-responsive mammary epithelial progenitors that are associated with breast cancer risk. Here, the authors find that short-term TGFBR1 inhibition prevents tumour formation in rat breast cancer models and identify a TGFBR1 inhibition-responsive sub-population of mammary epithelial cells, which is associated with human breast cancer risk.

## Introduction

The design of cancer-preventive strategies requires in-depth knowledge of physiological processes underlying tumor initiation, biomarkers to identify high-risk individuals and monitor the efficacy of cancer-preventive interventions, and approaches that effectively decrease risk with minimal side effects^1^. The best-known predictors of breast cancer risk are germline predispositions, reproductive history, and mammographic density^2^. A single full-term pregnancy in early adulthood reduces the lifelong risk of estrogen receptor-positive (ER+) postmenopausal breast cancer by almost twofold^3^, while high mammographic density increases risk regardless of tumor subtype^4^. Although anti-estrogens have been effective in preventing ER+ breast tumors, the associated side effects make their use unacceptable in the general population^5^. Similarly, using a full-term pregnancy in early adulthood as a cancer-preventive strategy is unrealistic. Instead, a better understanding of pregnancy-induced changes in the mammary epithelium may help the design of cancer-preventive strategies.

We previously described the lower expression of *CDKN1B* (encoding p27) and TGFβ signaling in CD44^+^ progenitor-enriched cells from normal breast tissue from parous compared to nulliparous women^6^. The relative frequencies of p27^+^ and Ki67^+^ (proliferating) cells were also lower in parous women, except in *BRCA1* mutation (*BRCA1*^*mut*^) carriers, who exhibited the highest fraction of these cells among all groups. Many p27^+^ cells were also ER^+^ and phospho-SMAD2^+^ (a downstream mediator of TGFβ signaling), with frequencies varying during the menstrual cycle and pregnancy, implying regulation by ovarian hormones and TGFβ signaling^6^. Based on these findings, we hypothesized that TGFβ is a key regulator of proliferation and pool size of hormone-responsive mammary epithelial progenitors that may serve as the cell-of-origin of breast cancer, and that decreasing these progenitors could reduce the rate of mammary tumor initiation.

The role of the TGFβ pathway in mammary gland biology and tumor development has been studied in mouse models by modulating the main components of the signaling cascade^7^. Cellular response to TGFβ is initiated by a ligand (TGFβ1, TGFβ2, or TGFβ3) binding to TGFBR2, a serine–threonine protein kinase receptor, which then phosphorylates and activates TGFBR1, the main downstream signaling receptor^8^. TGFBR1, also a serine–threonine protein kinase receptor, phosphorylates and activates the SMAD2 and SMAD3 transcription factors, which in turn mediate TGFβ-induced transcriptional changes^8^. Inhibition of TGFβ signaling has been shown to decrease mammary tumor growth in murine models of breast cancer through inhibiting cancer stem cells^9^. *Tgfb1* knockout mice in an immunodeficient background have normal mammary gland development, although there is a significant peripubertal decrease in the number of terminal end buds (TEBs), which are specialized structures leading the elongation of the invading duct and responsible for the formation of the mammary ductal tree^10^. The expression of dominant negative *Tgfbr2*^11^ or *Tgfbr2* deletion^12^ lead to alveolar hyperplasia in an epithelium-specific manner^13^ and accelerated MMTV-PyMT-induced tumor development^12^, while exogenous administration of TGFβ1 reversibly inhibits mammary epithelial cell proliferation^14^. Despite this knowledge, the cellular targets of TGFβ in the mammary epithelium have not been characterized in detail. Engineered murine models target specific cells in which the promoter expressing the transgene is active; thus, they are not suitable for cancer prevention studies that are agnostic to the cell-of-origin of cancer. Furthermore, most murine mammary tumors are ER-negative and estrogen-independent, and therefore not representative of hormone-dependent ER+ breast cancers, the most common subtype in women.

Here, we show that short-term treatment with galunisertib (LY2157299), a small molecule inhibitor of TGFBR1 (TGFBRi)^15^, reduces the frequency of mammary tumors induced by estrogen in inbred ACI rats^16^ and by NMU (N-Nitroso-N-methylurea) exposure in outbred SD rats^17^. We also identify a rare mammary epithelial cell subpopulation we term secretory basal cells (SBCs) that is sensitive to TGFBRi treatment in both rat strains and associated with breast cancer risk in women. These findings identify TGFβ signaling as a key determinant of breast cancer risk.

## Results

### The effect of TGFBR inhibitor treatment on peripubertal rats

**Fig. 1:**
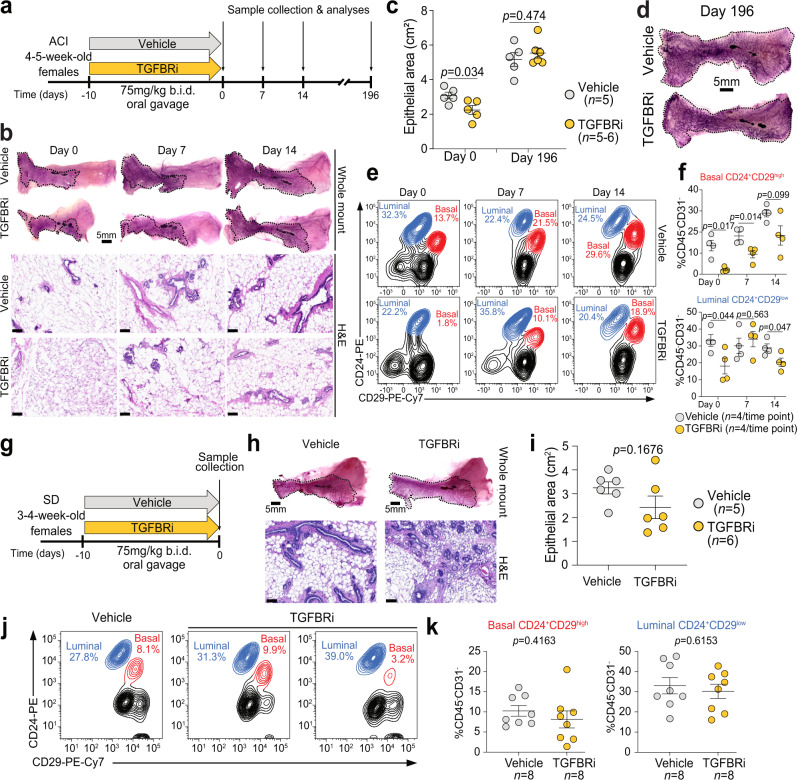
The effect of peripubertal TGFBRi treatment on mammary glands of peripubertal ACI and SD rats. **a** Schematic outline of experimental design for ACI rats. **b**–**d** Representative mammary gland whole mounts (**b**, **d**), quantification of the epithelial area (**c**), and hematoxylin and eosin (H&E) staining of mammary tissue sections (**b**) from ACI rats at indicated experimental timepoints (*n* = 5 for vehicle and for TGFBRi *n* = 5 at day 0 and *n* = 6 at day 196). **e**, **f** Representative flow cytometry plots (**e**) and quantification (**f**) of luminal (CD24^+^CD29^low^) and basal (CD24^+^CD29^high^) mammary epithelial cells within the CD31^−^CD45^−^ cell population in ACI rats. **g** Schematic outline of experimental design for SD rats. **h**, **i** Two representative mammary gland whole mounts (**h**) and quantification (**i**) of the mammary gland epithelium in SD rats. **j**, **k** Representative flow cytometry plots (**j**) and quantification (**k**) of luminal (CD24^+^CD29^low^) and basal (CD24^+^CD29^high^) mammary epithelial cells within the CD31^−^CD45^−^ cell population in SD rats. All graphs (**c**, **f**, **i**, **k**) are presented as mean ± s.e.m. *P* values were calculated by unpaired two-tailed *t* test with Welch’s correction. Scale bars: whole mounts 5 mm, H&E 100 µm. Source data are provided as a Source Data file.

To determine the effects of short-term inhibition of TGFβ signaling on the mammary epithelium, we treated 4–5-week-old virgin female ACI rats for 10 days with TGFBRi, and analyzed mammary glands at days 0, 7, 14, and 196 after stopping treatment (Fig. 1a). The timing and length of treatment were chosen based on the age known to be critical for mammary tumor susceptibility (peri-puberty), both in rats^18^ and in human^18^, and the duration of gestation in rats (21 days). TGFBRi treatment was well-tolerated and showed target inhibition as evidenced by lower levels of phospho-SMAD3, a transcriptional mediator of TGFβ signaling directly phosphorylated by TGFBR1^8^, in the mammary glands of TGFBRi-treated animals (Supplementary Fig. 1a–c). The total mammary epithelial area was significantly smaller (*P* = 0.034) on day 0 in TGFBRi-treated rats, but similar in size at day 196 post treatment, implying a transient delay with ductal development (Fig. 1b–d). Although ductal invasion during puberty is driven by TEBs, we did not detect visible differences in TEBs in whole mounts (Supplementary Fig. 1d), implying that either TGFBRi treatment induced subtle differences not readily identified by imaging or that the ductal invasion process remained unperturbed. Smooth muscle actin (SMA), a myoepithelial cell marker, demonstrated no discernable perturbations in mammary ductal structure and integrity (Supplementary Fig. 1e), and the relative frequency of proliferative (Ki67^+^ or phospho-histone H3^+^: pHH3) and apoptotic (cleaved caspase 3^+^) cells did not show consistent differences after TGFBRi treatment (Supplementary Fig. 1e–k). However, the relative fraction of CD24^+^CD29^high^ basal and CD24^+^CD29^low^ luminal mammary epithelial cell populations assessed by flow cytometry (Supplementary Fig. 1l) was significantly lower in TGFBRi-treated animals compared to controls at day 0 (*P* = 0.017 and *P* = 0.044, respectively), with less pronounced differences at later timepoints (Fig. 1e, f). These data establish that a 10-day TGFBRi treatment in peripubertal ACI rats is sufficient to temporarily reduce mammary epithelial cell numbers, with differences being most pronounced right after discontinuing treatment (day 0) and more significant in basal compared to luminal cells. Thus, we chose this time point and treatment schedule for subsequent experiments.

We also performed the above-described experiment in ~4-week-old virgin female SD rats (Fig. 1g) and again found that TGFBRi treatment was well tolerated (Supplementary Fig. 2a). Unlike in the ACI strain, at day 0, the total mammary epithelial area and the relative frequencies of basal and luminal cells were not significantly different in TGFBRi-treated animals as compared to controls, likely due to high interindividual differences in this outbred strain (Fig. 1h–k). In addition, no visible difference in the number of TEBs following TGFBRi treatment was observed (Supplementary Fig. 2b). The relative frequency of proliferative cells was also similar between treated and control groups based on Ki67 positivity while pHH3^+^ showed significant differences (Supplementary Fig. 2c–f).

To examine whether a 10-day TGFBRi treatment affects other organs and whether its effects on the mammary gland are due to perturbed puberty, we visually inspected all major organs, performed histologic analyses of pituitary glands, ovaries, intestinal tract, and endometrium, measured serum estrogen and progesterone levels, and assessed the frequencies of proliferative and hematopoietic stem cells. No significant differences were observed between vehicle and TGFBRi-treated animals in either rat strain in any of the parameters analyzed (Supplementary Fig. 3 and Supplementary Data 1).

Leukocytes play an important role in mammary gland development^19^, and TGFβ is an important regulator of both innate and adaptive immune cells^20^. Thus, we analyzed the mammary immune microenvironment in both rat models in all experiments performed. RNA-seq analysis of CD45^+^ leukocytes from mammary glands did not show any TGFBRi treatment-related changes in gene expression or cellular composition inferred using CIBERSORT^21^ at any time point and age analyzed (Supplementary Fig. 4a–f and Supplementary Data 1). Macrophages are required for normal mammary gland development^22^ and have been proposed to participate in the development of the mammary epithelial stem cell niche^23,24^ and TEBs^25^. Thus, we assessed the frequencies and location of CD163^+^ macrophages in mammary glands of TGFBRi-treated and control animals, but did not observe any significant differences (Supplementary Fig. 4g, h and Supplementary Data 1).

Taken together, these data indicate that short-term peripubertal TGFBRi treatment has the most pronounced effects on the mammary epithelium, although impacts on stromal and immune cells not detected by the approaches we used cannot be excluded.

### The effect of TGFBRi on mammary glands of adult ACI rats

Breast cancer prevention strategies are most likely to be tested first in high-risk women after their child-bearing years. Thus, we evaluated the effects of the TGFBRi treatment on the mammary glands of adult (18–24-week-old) virgin and parous ACI rats (Supplementary Fig. 5a). We found that mammary glands of TGFBRi-treated animals were visually and histologically normal (Supplementary Fig. 5b). However, the fraction of proliferative mammary epithelial cells (pHH3^+^) was significantly lower after TGFBRi treatment in all (virgin and parous combined) animals (*P* = 0.007), and in virgin animals alone (*P* = 0.029) (Supplementary Fig. 5c). In addition, the fraction of apoptotic (cleaved caspase 3^+^) cells was significantly higher (*P* = 0.035) in virgin animals after TGFBRi treatment (Supplementary Fig. 5d, e). Consistent with these findings, we detected a significant decrease in the relative frequency of CD24^+^CD29^high^ basal cells by flow cytometry in both virgin TGFBRi-treated animals (*P* = 0.032), as well as virgin and parous combined (*P* = 0.001), compared to controls, while the fraction of CD24^+^CD29^low^ luminal cells was not different (Supplementary Fig. 5f, g). These results demonstrate that TGFBRi also affects mammary epithelial cells in adult rats, more noticeably in virgin as compared to parous. However, similar to humans^18^, peripubertal animals seem to be more sensitive to factors affecting the mammary epithelium.

### TGFBRi treatment prevents NMU-induced mammary tumors in SD rats

**Fig. 2:**
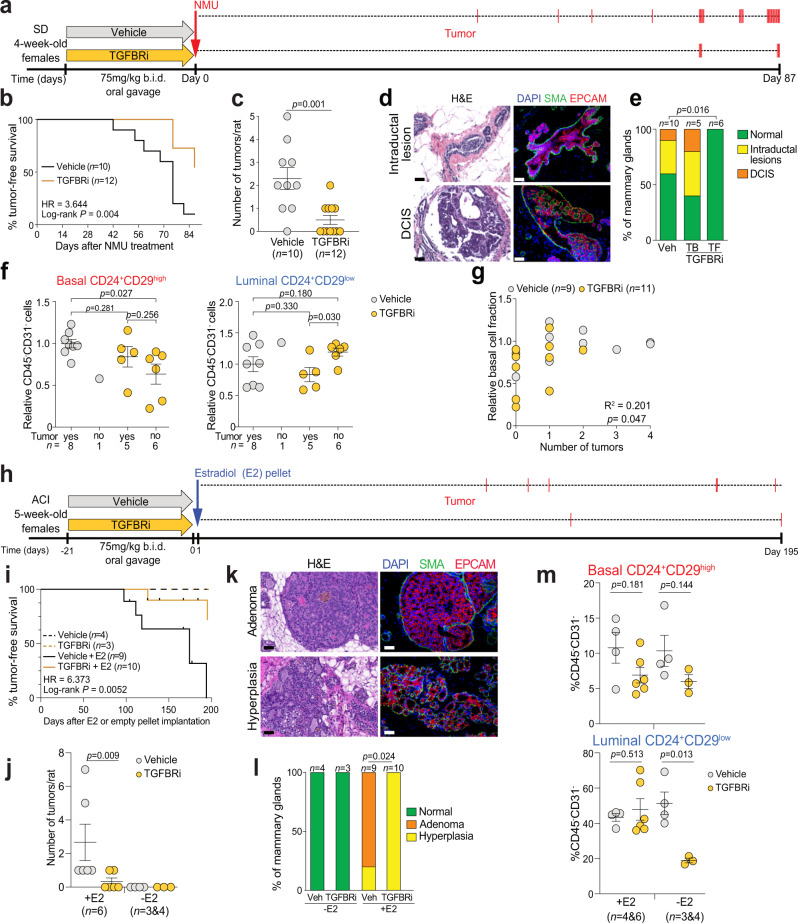
Peripubertal TGFBRi treatment prevents mammary tumor initiation. **a** Schematic outline of experimental design of NMU-induced tumors in SD rats. Red bars indicate the detection of a new tumor. **b** Kaplan–Meier tumor-free survival plot for SD rats. **c** Tumor burden per SD rat. **d**, **e** Representative images (**d**) and quantification (**e**) of microscopic lesions in mammary glands without palpable tumors. **f** Quantification of basal (CD24^+^CD29^high^) and luminal (CD24^+^CD29^low^) mammary epithelial cells within the CD31^−^CD45^−^ cell population from flow cytometric analysis in SD rats. **g** Relationship between the relative fraction of basal cells and tumor burden for all animals. **h** Schematic outline of experimental design of E2-induced tumors in ACI rats. Red bars indicate the detection of a new tumor. **i** Kaplan–Meier tumor-free survival plot for ACI rats. **j** Tumor burden per ACI rat. **k**, **i** Representative H&E and immunofluorescence images of SMA and EPCAM staining (**k**) and quantification (**l**) of histology in mammary glands without macroscopic tumors. **m** Quantification of basal (CD24^+^CD29^high^) and luminal (CD24^+^CD29^low^) mammary epithelial cells within the CD31^−^CD45^−^ cell population by flow cytometric analysis in ACI rats. All graphs (**c**, **f**, **j**, **m**) are presented as mean ± s.e.m. *P* values were calculated by log-rank test (**b**, **i**); one-sided Wilcoxon rank-sum test with continuity correction (**c**, **j**); Fisher’s exact test (**e**, **l**); unpaired two-tailed *t* test with Welch’s correction (**f**, **m**); linear regression model with the goodness of fit (*R*^2^) and *P* value (**g**). Scale bars: 50 µm. Source data are provided as a Source Data file.

We next investigated whether a short-term peripubertal TGFBRi treatment would prevent mammary tumorigenesis by treating 4-week-old female virgin SD rats with TGFBRi as described above followed by a single NMU injection on day 0 (Fig. 2a). Animals were sacrificed at the experimental endpoint, 87 days after NMU injection. We found that significantly fewer of the TGFBRi-treated rats developed palpable tumors compared to vehicle-treated controls (5/12 treated vs. 9/10 control, *P* = 0.0189, chi-square test) (Fig. 2a, b). We also observed a significant increase in latency of tumor development (hazard ratio 3.644, *P* = 0.004) in TGFBRi-treated rats compared to controls (Fig. 2b), together with a significant decrease in tumor burden (2.3 tumors/rat in vehicle vs. 0.5 in TGFBRi-treated animals, *P* = 0.001) (Fig. 2c). However, tumors in control and TGFBRi-treated rats did not exhibit significant differences (Supplementary Fig. 6a–l and Supplementary Data 2).

Interestingly, we observed significant histologic differences (*P* = 0.016) in the grossly normal-appearing mammary glands between TGFBRi- and vehicle-treated animals. While 4/10 animals in the vehicle and 3/5 in the TGFBRi-treated tumor-bearing groups had histologic abnormalities such as intraductal proliferative lesions resembling ductal hyperplasia and ductal carcinoma in situ (DCIS), 6/6 tumor-free animals harbored histologically normal mammary glands (Fig. 2d, e). Flow cytometry analysis of mammary glands showed a significant decrease (*P* = 0.027) in CD24^+^CD29^high^ basal epithelial cells in TGFBRi-treated animals that did not develop tumors relative to control tumor-bearing animals (Fig. 2f and Supplementary Fig. 6m). However, the frequency of Ki67^+^ mammary epithelial cells was not significantly different between tumor-bearing or tumor-free TGFBRi-treated and control animals (Supplementary Fig. 6n, o), and the relative fraction of basal cells showed weak correlation with tumor burden (*R*^2^ = 0.21, *P* = 0.047, Fig. 2g). These data imply that short-term TGFBRi treatment in peripubertal animals may induce lasting changes relevant to mammary tumor initiation.

### TGFBRi treatment prevents estradiol-induced mammary tumors in ACI rats

We next evaluated whether TGFBRi treatment could also prevent estrogen-induced tumorigenesis in ACI rats, which, when implanted with slow-release estradiol (E2) pellets after puberty (at 9 weeks of age) to avoid interference with pubertal development, develop ER + mammary tumors with a latency of about 6 months^16^. Thus, 5-week-old virgin female ACI rats were treated with TGFBRi for 21 days, followed by implantation of empty or E2 pellets (Fig. 2h). Animals implanted with empty pellets did not develop mammary tumors. In the E2-treated groups, we observed a significant increase in tumor-free survival (hazard ratio 6.373, *P* = 0.0052) with fewer TGFBRi-treated animals developing tumors (2/10) compared to controls (6/9) (Fig. 2i) and fewer mammary tumors per animal (2.66 in vehicle vs. 0.33 in TGFBRi-treated animals, *P* = 0.009; Fig. 2j). E2 also triggered pituitary adenomas irrespective of TGFBRi treatment (Supplementary Fig. 7a): 3/9 controls and 4/10 TGFBRi-treated rats developed fatal hemorrhagic pituitary adenomas, succumbing to them before the end of the experiment, and were censored in our survival analysis (Fig. 2i).

Similar to our findings in SD rats, tumors from TGFBRi-treated and vehicle control groups were histologically and molecularly similar, although the sample size in TGFBRi-treated animals was very small (only two tumors) (Supplementary Fig. 7b–f). Histologic analysis of visibly tumor-free mammary glands showed epithelial hyperproliferation resembling gestational hyperplasia in all E2-treated rats due to prolonged E2 treatment (Fig. 2k). Microscopic adenocarcinomas were only observed in vehicle-treated rats (8/9 rats), while TGFBRi-treated animals had no proliferative mammary epithelial lesions (Fig. 2k, l). Mammary glands showed no significant differences in the relative frequencies of basal cells in the TGFBRi-treated animals compared to controls implanted with E2-containing pellets. However, TGFBRi-treated animals implanted with empty pellets had a significant reduction (*P* = 0.013) in the relative fraction of luminal cells (Fig. 2m and Supplementary Fig. 7g), although the relative frequency of Ki67^+^ cells was not different among any of the groups analyzed (Supplementary Fig. 7h, i). E2-induced pseudopregnant mammary milk protein production was not affected by TGFBRi treatment (Supplementary Fig. 7j). These data suggest that short-term peripubertal TGFBRi treatment has a lasting cancer-preventive effect without perturbing normal mammary physiology in ACI rats.

### TGFBRi-induced changes in gene expression profiles

To dissect the molecular mechanisms underlying the cancer-preventive effects of TGFBRi treatment in rat mammary glands, we performed RNA-seq of FACS-purified CD24^+^CD29^high^ basal and CD24^+^CD29^low^ luminal normal mammary epithelial cells collected from peripubertal rats at variable timepoints after 10 days of TGFBRi treatment. Principal component analysis (PCA) and differentially expressed gene (DEG) analysis of ACI rat samples collected on days 0, 7, and 14 after stopping TGFBRi treatment demonstrated variability among samples, and day 0 samples showed the most TGFBRi treatment-related differences in both cell types (Supplementary Fig. 8a, b and Supplementary Data 3). MetaCore^26^ analysis of DEGs for enriched networks revealed the most significant enrichment in luminal cells at day 0 with a significant decrease in cell cycle-related networks (Supplementary Fig. 8c and Supplementary Data 4). In young SD rats, we identified very few DEGs between vehicle and TGFBRi-treated animals, likely due to high inter-animal variability (Supplementary Fig. 8d and Supplementary Data 3). In adult ACI rats, the most pronounced TGFBRi treatment-associated gene expression changes were observed in basal cells of virgin animals, with downregulation of many cell adhesion and extracellular matrix-related networks (Supplementary Fig. 8e–g and Supplementary Data 3 and 4).

We also analyzed basal and luminal epithelial cells from normal mammary glands of NMU-treated SD, and E2-treated ACI rats terminated at the end of the tumor experiments (87 and 195 days after stopping TGFBRi treatment in ACI and SD rats, respectively) to identify TGFBRi-induced persistent changes. The most pronounced differences were observed in luminal cells between the tumor-free TGFBRi-treated and vehicle groups of SD rats, with DEGs showing significant enrichment in cell adhesion, immune, signaling, and transcription/translation networks (Supplementary Fig. 8h–m and Supplementary Data 3 and 4). Overall, these gene expression changes are consistent with the inhibition of TGFβ signaling as many known TGFβ targets were downregulated, and they also demonstrate that short-term TGFBRi treatment induces persistent phenotypic differences in the mammary epithelium.

### Characterization of the rat mammary gland at single-cell resolution

To dissect TGFBRi-induced cellular and molecular changes in the mammary epithelium in further detail, we performed single-cell RNA-sequencing (scRNA-seq) on FACS-purified CD24^+^CD29^high^ basal and CD24^+^CD29^low^ luminal cells from peripubertal ACI and SD rats, immediately following the 10-day TGFBRi regimen (D0). In total, we analyzed 17,000 cells from ACI rats with 3 animals per treatment group, and 40,000 cells from SD rats, with 3 control and 6 TGFBRi-treated animals. To eliminate any potential stromal contamination, a keratin filter was employed to classify cells with at least 2 reads aligning to any keratin as epithelial (Supplementary Fig. 9a). Epithelial cells from TGFBRi-treated SD rats had overall higher keratin levels compared to controls, suggesting that TGFBRi treatment promotes epithelial features (Supplementary Fig. 9b).

**Fig. 3:**
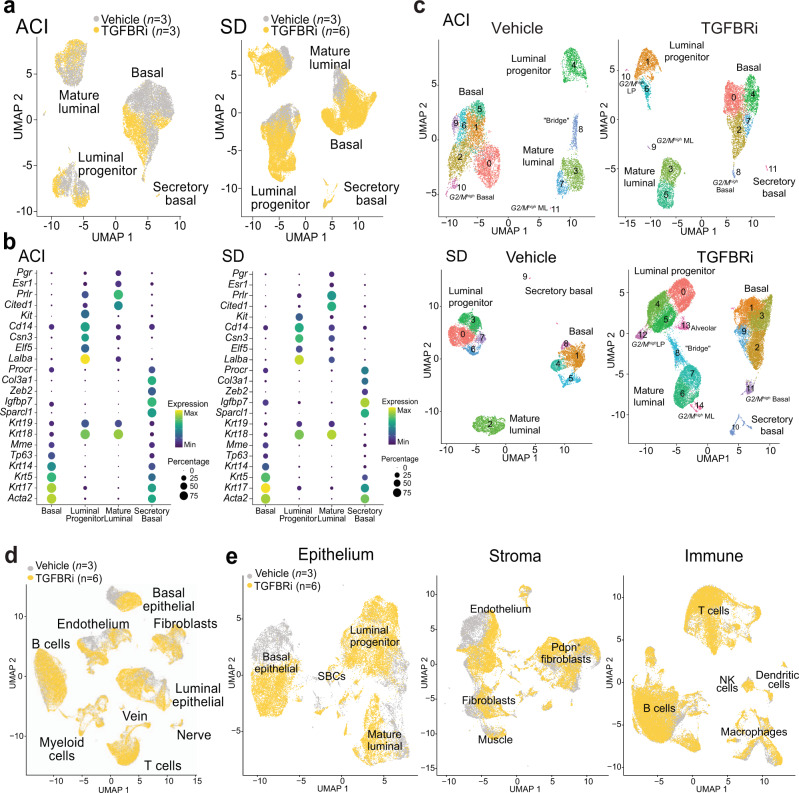
Single-cell RNA-sequencing analysis of mammary epithelial cell populations. **a**, **b** UMAP plots (**a**) and dot plots (**b**) of sorted basal (CD24^+^CD29^high^) and luminal (CD24^+^CD29^low^) mammary epithelial cell fractions from ACI and SD rats treated with vehicle and TGFBRi. Dot plots show the expression of markers representative of the four identified cell types. **c** Integrated UMAP plots of all cells from the vehicle and TGFBRi-treated ACI and SD rats. **d**, **e** UMAP plots of whole mammary gland scRNA-seq data from SD rats preprocessed and analyzed together (**d**) or separately for each major cell type (**e**). Cells are colored by treatment condition (**a**, **d**, **e**) or by cluster assignment (**c**).

Consistent with previous scRNA-seq reports on human and mouse mammary epithelium^27–29^, we identified three major cell populations: basal, luminal progenitor (LP), and mature luminal (ML) cells (Fig. 3a, b and Supplementary Fig. 9c). In addition, we detected a unique rare subpopulation present in both SD (849 cells, 2.1% of all cells) and ACI rats (48 cells, 0.28% of all cells) that we termed secretory basal cells (SBCs) due to their basal phenotype and high expression of secreted proteins (e.g., *Sparcl1*, *Igfbp7*). The SBC subpopulation was enriched after TGFBRi treatment, consisting of 91% (SD) and 77% (ACI) TGFBRi-treated cells. Cells segregated mostly by treatment in both strains (Fig. 3a), with samples from the more homogenous ACI inbred strain mixing well within each treatment condition, and samples from the more heterogeneous SD outbred strain displaying animal-driven clustering (Supplementary Fig. 9d). Key TGFβ signaling components were expressed to varying degrees across cell types, with basal cells and SBCs having highest expression of *Tgfbr3* compared to luminal cells in both strains, which implies their higher sensitivity to TGFβ (Supplementary Fig. 9e, f).

By analyzing each of the two treatment conditions separately and via integration of cells across animals^30^ (see “Methods”), we confirmed the existence of the four main epithelial populations in both strains (Fig. 3c and Supplementary Fig. 9f). We also observed multiple smaller subpopulations depicted as “tails” of major cell types, and as a transitional “bridge” between LP and ML cells (Fig. 3c). In ACI rats, some of these subpopulations were already present at low levels in vehicle-treated animals, while in SD rats, they were specific to TGFBRi-treated samples. These “tails” were identified as either cell expressing G2/M cell cycle phase markers (G2/M^high^) or progenitor-like cells expressing alveolar markers (*Elf5*), and transcription factors inhibiting differentiation (*Id1* and *Id2*) (Supplementary Data 5).

TGFβ is a pleiotropic cytokine that affects many cell types. Thus, to explore how TGFBRi treatment may affect other mammary cellular populations besides the epithelium, we repeated the scRNA-seq experiment using whole mammary glands from SD rats. In total, we analyzed 110,000 cells from 3 control and 6 TGFBRi-treated animals. We identified all major cell types (epithelial, stromal, and immune) based on distinct expression of cell type-specific markers and we observed good mixing of the samples in each cell type (Fig. 3d and Supplementary Fig. 10a–d). We detected a clear TGFBRi treatment effect in basal and luminal mammary epithelial cells, and in the endothelium (Fig. 3e). To characterize the affected cell populations in further detail, we performed an in-depth analysis of each major cell type (Supplementary Data 6). Within the epithelium, basal cells showed the most pronounced difference between vehicle and TGFBRi groups (Fig. 3e and Supplementary Fig. 10e), consistent with our findings from bulk RNA-seq and sorted scRNA-seq. In the stroma, all cell types (endothelium, muscle, and fibroblasts) showed treatment-related differences, while in the immune cell populations, only macrophages showed a TGFBRi-specific response (Fig. 3e and Supplementary Fig. 10f, g). Analysis of the differentially expressed genes (Supplementary Data 6) within the affected cell types by GSEA demonstrated significant enrichment for pathways related to TGFβ signaling, including SMAD binding and extracellular matrix and developmental processes (Supplementary Fig. 10h).

Overall, our scRNA-seq data in both sorted epithelium and whole mammary gland showed that the TGFBRi treatment-induced changes most likely to impact mammary tumor initiation are most pronounced in the mammary epithelium.

### Secretory basal cells: a unique subpopulation associated with breast cancer risk

**Fig. 4:**
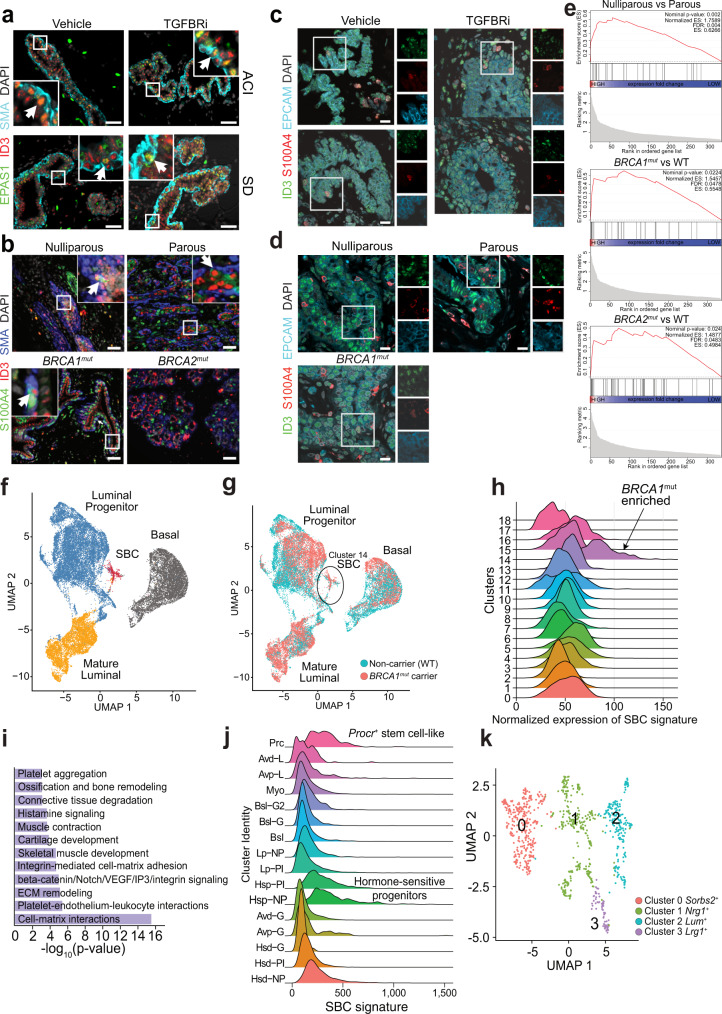
Secretory basal cells in mammary gland and tumor development. **a**, **b** Representative immunofluorescence images of EPAS1/HIF2A, ID3, and SMA in ACI and SD mammary glands (**a**) and of S100A4, ID3, and SMA in normal breast tissue sections from noncarrier parous (*n* = 2) and nulliparous (*n* = 3) women, as well as from *BRCA1/2* mutation carriers (*n* = 6) (**b**). Scale bar 50 μm. **c**, **d** Representative immunofluorescence images of ID3, S100A4, and EPCAM in SD rat mammary glands (**c**) and in normal breast tissue sections from noncarrier parous (*n* = 2) and nulliparous (*n* = 2) women, as well as from *BRCA1* mutation carriers (*n* = 2) (**d**). Scale bar 10 μm. **e** GSEA plots showing the enrichment of the SBC signature in DEGs within the indicated comparisons. *P* values are calculated using Kolmogorov–Smirnov test following the Benjamini–Hochberg adjustment. **f**, **g** UMAP plots of normal human breast scRNA-seq data^31^ colored by assigned cell type (**f**) or by *BRCA1* mutational status (**g**). **h** Ridge plot showing the expression of the SBC signature in the indicated clusters of human mammary epithelial scRNA-seq data^31^. **i** MetaCore enrichment on gene sets characteristic to SBCs. *P* values are calculated by hypergeometric test. **j** Ridge plot showing the expression of the SBC signature in the indicated clusters of mouse mammary epithelial scRNA-seq data^28^. **k** UMAP plot depicting the SBC subclusters in SD rats colored by cluster assignment.

We then analyzed the SBC cell population in further detail to better understand their role in mammary gland biology and breast cancer risk. We defined a SBC signature consisting of genes differentially expressed in SBCs compared to basal cells in both strains (e.g., *Id3, S100a4*, and *Epas1*) (Supplementary Fig. 11a, b and Supplementary Data 7). Analysis of the SBC signature in our whole mammary gland scRNA-seq data revealed high expression in fibroblasts and endothelial cells in addition to a subset of basal epithelium, which is expected due to the high expression of stem cell related and mesenchymal markers in SBCs (Extended Data Fig 11c). Thus, we performed multicolor immunofluorescence for SBC and epithelium-specific (e.g., EPCAM) markers to confirm their epithelial identity and presence in the mammary ducts. We observed a rare ID3^+^EPSA1^+^SMA^+^ basal population by immunofluorescence in mammary glands of peripubertal rats (Fig. 4a). Similarly, using normal breast tissues from women of different parity (i.e., nulliparous and parous) and mutational status (6*BRCA1*^*mut*^ and 3 *BRCA2*^*mut*^), we identified a rare epithelial cell population that was ID3^+^S100A4^+^SMA^+^ within all samples (Fig. 4b). However, the frequency of these cells was too low (<1%) for reliable quantification and comparison between groups despite scanning the entire slide (>5000 epithelial cells). We also detected rare EPCAM^+^ cells within the mammary epithelium clearly positive for SBC markers, confirming the epithelial identity of SBCs in both the rat mammary gland and in normal human breast tissues (Fig. 4c, d).

We further investigated potential associations between SBCs and breast cancer risk by assessing the enrichment of the SBC signature in sets of genes differentially expressed in CD44^+^ progenitor-enriched cells between nulliparous relative to parous women and *BRCA1/2*^*mut*^ carriers relative to non-carriers^6^. The SBC signature was significantly enriched in samples with higher breast cancer risk (i.e., nulliparous, and *BRCA1/2*^*mut*^ carriers) compared to those of lower risk (FDR < 0.05 for all cases, Fig. 4e). Similarly, by analyzing published scRNA-seq data of normal breast tissues from women with different risks of breast cancer^31^, we confirmed the existence of a distinct minor subpopulation with high expression of SBC markers as a tail of the luminal progenitor cluster enriched in *BRCA1*^*mut*^ samples (60% of SBC cells were *BRCA1*^*mut*^ versus 44% of all other cells, chi-squared *P* = 5 × 10^−9^) (Fig. 4f–h and Supplementary Fig. 11d–f).

### SBCs and mammary epithelial cell hierarchy

Next, we investigated the biological functions of SBCs, focusing on SD rats, in which this population was more prominent. The SBC signature was enriched in the extracellular matrix, TGFβ signaling, response to estradiol, progenitor, and stem cell functions (Fig. 4i and Supplementary Fig. 11g, h). Analysis of published mouse mammary gland scRNA-seq data^28^ revealed SBC enrichment in Procr-positive (*Procr*^*+*^ stem cell-like) and hormone-sensitive progenitor (Hsp) subpopulations (Fig. 4j).

Further clustering of the SD SBCs alone revealed four subclusters with distinct gene expression profiles (Fig. 4k, Supplementary Fig. 11i, and Supplementary Data 7). Interestingly, the *Sorbs2*^*+*^ and *Lrg1*^*+*^ subclusters expressed *Procr*^32,33^ and *Cdh5*^34^, respectively, implicated in mammary stem cells. Since *Procr*^*+*^ cells were previously described as pluripotent mammary epithelial stem cells^32,33^, we investigated the relatedness of our SBCs to mouse *Procr*^*+*^ cells in further detail. GSEA showed positive enrichment of mouse Procr^+^ cell-specific genes in our SBC signature, while genes enriched in Procr^neg^ cells also showed enrichment in our basal non-SBC cells (Supplementary Fig. 11j). The same pattern was observed when we analyzed each SBC subcluster individually (Supplementary Fig. 11k). These data suggest that SBCs represent a mixed cell population that include Procr^+^ mammary epithelial stem cells.

**Fig. 5:**
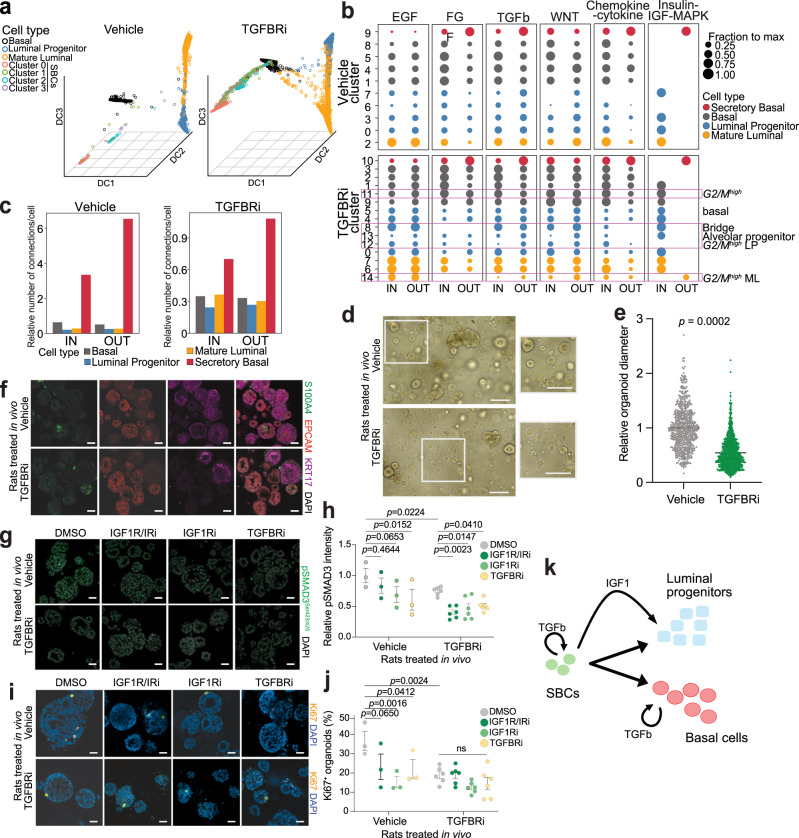
Functional relevance of TGFBRi-induced changes in the mammary epithelium. **a** Diffusion maps of mammary epithelial cells from control and TGFBRi-treated SD rats. **b** Cell type-specific inferred connectivity among selected signaling pathways from interactome analysis in SD control and TGFBRi-treated rats. Clusters refer to the integrated data in Fig. 3c, with selected clusters highlighted. The size of dots is the fractional number of either IN or OUT connections per cluster per pathway, normalized across clusters. IN—incoming (target); and OUT—outgoing (source) signals. **c** Relative number of inferred interactome connections per cell, aggregated by cell type. **d** Representative images of organoid cultures from the vehicle and TGFBRi-treated SD rats. **e** Plot depicting quantification of relative organoid diameter. Organoids were derived from animals treated with vehicle (*n* = 3) or TGFBRi (*n* = 6). Two wells/animal were used for quantification. **f** Representative immunofluorescence images of organoids for S100A4, EPCAM, and KRT17. Immunofluorescence was performed once on multiple samples from vehicle (*n* = 3) and TGFBRi (*n* = 5) treated rats. **g**–**j** Representative immunofluorescence images (**g**, **i**) and quantification (**h**, **j**) of organoids for pSMAD3 and Ki67^+^ following treatment with the indicated inhibitors. Organoids were derived from animals treated with vehicle (*n* = 3) or TGFBRi (*n* = 6), then treated with the indicated inhibitors in vitro. Two wells/animal/treatment were used for quantification. **k** Schematic model of TGFBRi treatment effects on mammary epithelium relevant to cancer prevention. Scale bars: organoids 100 µm, immunofluorescence 20 µm. Graphs (**e**, **h**, **j**) are presented as mean ± s.e.m. *P* values were calculated by unpaired two-tailed *t* test (**e**) and by two-way ANOVA (**h**, **j**). ns denotes not significant. Source data are provided as a Source Data file.

Next, we delineated the pseudotime-based differentiation trajectory of the mammary epithelial cell types using Monocle3^35^, and defined SBCs as the starting point in TGFBRi-treated samples and *Tp63*^+^ basal cells in controls due to the rarity of SBCs in these samples and the essentiality of *Tp63* for mammary epithelial development^36^ (Supplementary Fig. 12a–c). The pseudotime trajectory in the TGFBRi-treated samples extended from SBCs to basal cells, continuing from *Egr1*^−^ ML cells to *Egr1*^*+*^ ML cells, and further from *Apoe*^+^ LP cells to *Apoe*^−^ LP cells, reinforcing the fact that SBCs are most closely related to *S100a6*^+^ basal cells. In addition, inferred diffusion maps^37^ of the vehicle and TGFBRi-treated animals were strikingly distinct (Fig. 5a). In drug-treated animals, the basal cells formed a continuous bridge connecting the SBC subclusters with luminal cells, with the *Nrg1*^*+*^ SBC cluster 1 representing the population closest to the basal cells, and the SBC *Sorbs2*^*+*^ *Procr*^*high*^ cluster 0 furthest apart from the basal.

To assess paracrine interactions among SBCs and other mammary epithelial cells, we performed interactome analysis on the integrated scRNA-seq data with all cell types in SD rats, separately for the vehicle and TGFBRi-treated samples (Fig. 5b and Supplementary Data 5) and using signaling pathways with key roles in mammary epithelial differentiation (Supplementary Data 8). The inferred cell–cell connections among clusters acting as either the source or target of signaling indicated strong cell type-specific differences in the relative activity of the analyzed signaling pathways. FGF, TGFβ, WNT, chemokine-cytokine, interleukin, Notch, and axon-guidance signaling pathways were enriched in basal relative to luminal cell types, while EGF and incoming insulin-IGF-MAPK signaling were more active in luminal cells (Fig. 5b and Supplementary Fig. 12d). Among all cell types, SBCs showed the highest relative number of connections per cell (Fig. 5b, c and Supplementary Fig. 12e) and consistently had more outgoing than incoming connections for the majority of signaling pathways, including FGF, TGFβ, chemokine-cytokine, and insulin-IGF-MAPK in both treatment conditions (Fig. 5c and Supplementary Fig. 12d–f). The insulin-IGF-MAPK signaling pathway displayed a special pattern of cell type-specific communication: SBCs were the almost exclusive source, while basal and luminal cells acted almost exclusively as targets. The only prominent signaling pathway with higher incoming than outgoing activity in SBCs was WNT signaling with well-established roles in self-renewal and maintenance of progenitor cellular phenotypes (Supplementary Fig. 12e). These data demonstrate a central role for SBCs in the mammary epithelium since insulin-IGF signaling is one of the most critical pathways regulating both pubertal mammary gland development and breast cancer risk^38^.

### TGFBRi treatment lastingly reduces mammary epithelial cell proliferative potential

To experimentally test the predictions of our molecular and functional network analyses, we derived organoid cultures from peripubertal SD rats 10 days after vehicle or TGFBRi treatment. Organoids derived from TGFBRi-treated animals were significantly smaller in size compared to vehicle-treated ones, even when grown in the same culture conditions, suggesting persistent reduction in proliferative capacity due to TGFBRi treatment (Fig. 5d, e). Importantly, we confirmed the presence of SBCs in organoids by immunofluorescence for SBC and epithelial markers, again identifying them as a rare epithelial subpopulation (Fig. 5f and Supplementary Fig. 12g).

Next, we treated organoids with inhibitors of TGFBR1 or IGF1R for 8 days and assessed cell proliferation and signaling pathway activity by immunofluorescence for Ki67 and phospho-SMAD3 (pSMAD3)/phopho-S6 (pS6), respectively. Organoids derived from TGFBRi-treated animals had significantly fewer Ki67^+^ and pS6^+^ cells, as well as reduced pSMAD3 intensity, even at baseline compared to those derived from vehicle controls, and this growth-suppressive effect was further accentuated following in vitro treatment with IGF1R or TGFBR inhibitors (Fig. 5g–j and Supplementary Fig. 12h, i). We found that the fraction of pS6^+^ cells was decreased following both IGF1R and TGFBR inhibitor treatment, and similarly IGF1R inhibitor-treated organoids had decreased pSMAD3 intensity, demonstrating crosstalk between IGF1R and TGFβ signaling.

These data show that TGFBRi treatment has a direct cell-autonomous effect on mammary epithelial cells, inducing lasting changes that make them less proliferative even after treatment is discontinued.

## Discussion

Stem cells and progenitors are the cell-of-origin of cancer^39^, and thus, targeting them could be an effective cancer prevention strategy^40^. Building on our prior data^6,41^, here we tested the hypothesis that short-term inhibition of TGFβ signaling prevents mammary tumors in rat models of breast cancer. We found that short-term TGFBRi treatment of peripubertal inbred ACI and outbred SD rats induced lasting changes in the mammary epithelium and significantly reduced mammary tumorigenesis induced by estrogen and NMU, respectively. Cellular and molecular analyses of TGFBRi treatment effects showed significant changes in basal and luminal mammary epithelial cells and identified a unique subpopulation with progenitor features, termed SBCs, which expands after TGFBRi treatment. SBCs have high levels of several markers associated with mammary stem cells^28,33,34,42,43^. A newly derived SBC gene signature showed enrichment in genes highly expressed in nulliparous versus parous women, as well as in *BRCA1/2*^*mut*^ carriers versus non-carriers. Thus, TGFBRi treatment affects cells relevant for mammary differentiation and cancer in both rats and humans.

Mammary gland development occurs mostly postnatally during puberty, pregnancy, and lactation^44^, leading to changes in cellular composition and susceptibility to mammary tumors^25,45^. We observed an expansion of progenitor-like SBCs and a decrease in mammary tumor incidence in our rat models following TGFBRi treatment and higher expression of the SBC signature in normal progenitor-enriched cells from women with increased breast cancer risk (e.g., nulliparous, *BRCA1*^mut^). We formulated a working mechanistic model to explain these seemingly paradoxical observations (Fig. 5k). TGFβ enhances the growth of both basal cells and SBCs that might represent bipotential stem cells. Thus, treatment with TGFBRi decreases the relative fraction of both basal cells and luminal progenitors and leads to an increase in SBCs, possibly due to negative feedback loop from more differentiated cells. Despite being a rare subpopulation, SBCs are the most interactive cell type and the main source of insulin-IGF-MAPK signaling targeting luminal and basal cells. Insulin-IGF signaling is a key regulator of mammary epithelial progenitors, and higher activity of this signaling pathway increases human breast cancer risk^25,38,46^. SBCs may represent mammary epithelial stem cells that are stimulated by TGFβ and, due to their secretion of IGF1, enhance the proliferation and increase the pool size of hormone-responsive progenitors. Thus, women with more SBCs might be at higher risk of breast cancer, which is consistent with human epidemiological data demonstrating that pubertal IGF1 levels determine lifetime breast cancer risk^47^. These peripubertal TGFBRi treatment-triggered alterations in SBCs, and based on our prior data early full-time pregnancy^6^, cause a persistent decrease in progenitors with proliferative capacity, which results in a persistently lower risk of mammary tumor initiation. Our data showing reduced proliferation and lower pSMAD3^+^ and pS6^+^ levels of organoids derived from TGFBRi-treated animals and a decrease in proliferation and decrease in pSMAD3^+^ and pS6^+^ cells after both TGFBR or IGF1R inhibition are consistent with this model and indicate crosstalk between the two signaling pathways. Because we could not analyze the same animals both at the time of stopping TGFBRi treatment and at the end of the tumorigenesis assays, further studies are required to identify the cells from which tumors originate and their relationship to SBCs. In addition, it was not feasible to test if TGFBRi treatment during adulthood would also lead to a decrease in tumor incidence due to the resistance of adult animals to NMU/E2-induced tumorigenesis^48^.

In sum, our data provide preclinical evidence that breast cancer can be prevented by short-term inhibition of TGFβ signaling that induces lasting changes in the mammary epithelium and identifies markers for future translational studies aimed at risk prediction and prevention in women with a high risk of breast cancer.

## Methods

### Ethics statement

All clinical samples and data were collected following approval by Dana-Farber Cancer Institute Institutional Review Board (protocols 08–010, 10–458, 93–085, 14–400). All animal studies were conducted in accordance with the regulations formulated by the Dana-Farber Cancer Institute Animal Care and Use Committee (IACUC; protocol #15-005).

### Human breast tissue samples

All experiments with use of human breast tissue were approved by the Dana-Farber Cancer Institute Institutional Review Board (protocols 08–010, 10–458, 93–085, 14–400). Normal breast tissue samples were collected from women undergoing reduction mammoplasty or prophylactic mastectomy following written informed consent using protocols 08–010 and #10–458. Samples were de-identified in the tissue bank prior to transfer to the laboratory.

### Rat experiments and tissue harvesting

All animal experiments were performed in an AAALAC-accredited SPF rodent-only barrier facility at Dana-Farber Cancer Institute. All rats are housed in individually ventilated, solid-bottom, polysulfone 135 sq. in. microisolator cages. The cages are used in conjunction with the Optimice® rack systems with integrated automatic watering. Temperature and humidity in the rodent facility is controlled at 72+/− 2 °F and a target range of 35–55% relative humidity. A standard photoperiod of 12 h light/12 h dark is controlled by an automated system. All animal experiments were performed following protocol #15-005 approved by the Dana-Farber Cancer Institute Institutional Animal Care & Use Committee. Animals were euthanized by CO2 inhalation. Maximum tumor size burden allowed for rats is 3 cm, and this was not exceeded in any of the experiments. Four-week-old virgin female Hsd:Sprague Dawley SD (SD) female rats as well as 4- to 5-week-old virgin and 18- to 24-week-old virgin and parous ACI/SegHsd (ACI) female rats were purchased from Envigo.

LY2157299 (TGFBRi) was provided by Eli Lilly (Indianapolis, IN) and stored at −20 °C in powder form. LY2157299 was dissolved in a vehicle (1% carboxymethylcellulose, 0.5% sodium lauryl sulfate, 0.085% Povidone) for in vivo treatments and stored at 4 °C for up to 1 week. Animals were randomly allocated into the treatment groups. The sample size was chosen based on expected treatment effects to provide 80% power to draw conclusions. All rats were treated with vehicle or 75 mg/kg of LY2157299 by oral gavage twice daily (8–16 h between doses) for 10–21 consecutive days. Animal body weights were recorded every 2–3 days for all studies. For short-term treatment experiments, peripubertal animals were treated at about 24 (SD) or 35 days (ACI) of age, whereas postpubertal virgin and parous animals (ACI) were treated at 18–24 weeks of age. Peripubertal rats were sacrificed at days 0 (ACI and SD), 7 (ACI), 14 (ACI), and 196 (ACI) after the final LY2157299 dose. Postpubertal rats (ACI) were sacrificed at day 0 after the final LY2157299 dose.

For carcinogen-induced tumor experiments, SD rats were treated with vehicle or LY2157299 starting at 21 days of age for ten days, followed by a single intraperitoneal (i.p.) injection of 50 mg/kg N-nitroso N-methylurea (NMU) completion of the 20 doses/10-day LY2157299 regimen. NMU-treated SD rats were sacrificed 87 days later when control animals developed palpable tumors. ACI rats were treated twice daily with vehicle or LY2157299 starting at 38 days of age for 21 days. Animals were randomly allocated into the treatment groups. The sample size was chosen based on expected treatment effects and expected tumor incidence of the models to provide 80% power to draw conclusions. Upon completion of the TGFBRi treatment, 60-day-old rats were implanted with silastic implants containing air or estradiol (Sigma Aldrich, #E1024-25G). Briefly, implants were made in-house as described previously^49^ by sealing one end of the silastic tubing, filling it up with 37 mg of estradiol, and then sealing the other end of the tube with silicone. Silastic tubes containing air or estradiol were implanted in the rats’ neck region by making a 3-mm incision, detaching the skin, inserting the implant, and closing the wound with clips. Estrogen-treated and control animals were sacrificed 195 days later after all vehicle and estradiol-treated animals developed palpable tumors. Animals that developed poor health or poor body condition due to pituitary gland adenomas were sacrificed before the endpoint and were hence censored from the survival analysis. Kaplan–Meier survival analyses for both tumor experiments were performed by analyzing the dates when tumors became palpable using GraphPad Prism v8.

For all tumor experiments, after sacrificing the animals, tumor histology was examined to confirm malignancy. In addition, tumor incidence, multiplicity, size and/or weight were recorded. For some rats, mammary gland whole mounts were prepared of the inguinal/abdominal gland as described previously^50^. Briefly, the inguinal and abdominal mammary glands were collected, spread on glass microscope slides, fixed, stained in carmine, dehydrated, and cleared in xylene. Whole mounts of mammary glands were photographed with a dual 12MP wide-angle and telephoto camera. Images were analyzed using Fiji software (ImageJ 1.53)^51^ by measuring the total area occupied by the epithelium in each mammary gland. A 5mm-section of the inguinal/abdominal mammary gland was saved for histological analysis and subsequent molecular profiling by immunofluorescence. To this end, samples were fixed in 10% formalin overnight, stored in 70% ethanol, then paraffin-embedded (FFPE: formalin-fixed, paraffin-embedded) and processed into histological slides as described previously^52^. From the remaining fresh tissue (inguinal/abdominal + thoracic), single-cell suspensions for subsequent flow cytometry analysis, FACS, and sequencing were generated as described^52^. Briefly, tissues were digested in a collagenase solution (2 mg/ml collagenase type IV (Worthington, LS004189) in DMEM/F12 (Fisher Scientific) with constant stirring at 37 °C for 1–2 h followed by subsequent analyses or freezing viably in 10% DMSO/FBS. Bone marrow was collected (by flushing the femurs) and frozen viably in 10% DMSO/FBS for subsequent flow cytometry analysis. Intestines, ovaries, uterus, and pituitary glands were also collected from the peri-puberty rats and processed as described below for histological examination and/or immunofluorescence analysis. Pituitary glands were also collected from animals in the ACI tumor experiment. In addition, blood was collected by gravity by decapitating just-killed rats into microtainers from peripubertal animals and ACI rats in tumor experiment. Serum was isolated from blood and flash-frozen for subsequent hormone concentration measurements. For the tumor prevention experiments, tumors were also excised. A small slice of each tumor was saved for paraffin embedding, while the rest of the tumor was digested and frozen as described above for molecular profiling. If the tumor was too small for both procedures, then it was only fixed for histological analysis.

### Rat mammary organoid cultures

Rat mammary gland cultures were performed as previously described^53^. Briefly, mammary glands were collected and minced into 1–2-mm^3^ pieces, then transferred into digestion buffer (1 mg/mL collagenase IV, Sigma, 11088882001) and incubated on a shaker at 37 °C for 1 h. After digestion, the tissues were sheared using a 5 ml pipette and a bent P1000 tip. Then, the digestion was stopped by AdDF +++ medium (Advanced DMEM/F12 containing 1% GlutaMax, 1% 1 M HEPES, 1% penicillin–streptomycin and 0.1% Amphotericin B) with 2% FBS (Sigma, F2442). After centrifugation at 2000×*g*, the pellet was resuspended with 50 μl growth factor reduced (GFR) basement membrane matrix and placed on a 24-well culture plate to solidify at 37 °C for 30 min. After the “dome” structure was formed, 500 μl organoid culture medium (AdDF + ++medium containing B27, N-acetylcysteine, nicotinamide, R-spondin, FGF10, FGF7, Heregulin, EGF, and Y-27632) was added and then organoids were cultured in a 37 °C incubator. Organoids were passaged every 10 days using TrypLE digestion. At passage 3, the organoids were treated with 1 μM GSK1904529A (MCE, HY-10524), 1 μM Linsitinib (MCE, HY-10191) and 1 μM LY2157299 (MCE, HY-13226) for 8 days, respectively. Then, the organoids were collected for immunofluorescence analysis.

### Histology

In all, 4-micron FFPE tissue sections were deparaffinized and stained with hematoxylin and eosin (H&E) following standard procedures as described previously^52^. Normal mammary gland and mammary tumor histology was determined by an experienced rodent pathologist. H&Es of mammary glands were imaged using the Panoramic MIDI II digital slide scanner (3DHistech) and visualized and quantified using QuPath software^54^. The remaining brightfield images were acquired on a Nikon Ti/E inverted microscope using Nikon Elements software. Tumor cellularity was determined using H&E images. To this end, three random fields were imaged per tumor and QuPath software was used to detect the number and sizes of nuclei per image.

### Immunofluorescence and quantification

Multicolor immunofluorescence analyses were performed as described previously^52^. Briefly, after heat-induced antigen retrieval in sodium citrate (pH = 6) or TRIS-EDTA buffer (pH = 9), the samples were permeabilized with 0.5% Triton X-100, blocked with 100% goat serum, and stained with the respective antibodies. Rat mammary gland tissues were subject to immunofluorescence with antibodies (Supplementary Table 1) against pSMAD3, Ki67, pHH3, clCASP3, CD163, SMA, EPCAM, RAM milk proteins, ID3, and EPAS1 (HIF2A). Human normal breast tissues collected from patients who underwent breast reduction or prophylactic mastectomy were subject to immunofluorescence with antibodies against SMA, ID3, and S100A4. To allow multiplexing of these two rabbit IgG antibodies (ID3 and S100A4), the ID3 signal was detected using Tyramide Signal Amplification kit (Akoya Biosciences, SAT700001), whereas the S100A4 signal was detected by standard fluorophore-conjugated secondary antibody binding. Rat tumors were subject to immunofluorescence with antibodies against ER, PR, and pHH3. Intestines and endometrium were stained with antibodies against pHH3 and SMA. DAPI was used to stain nuclei. Stained slides were scanned using the Pannoramic MIDI II digital slide scanner (3DHistech) or imaged with a Nikon Ti/E inverted microscope using Nikon Elements software. pHH3 staining of mammary glands was quantified by selecting all the epithelium areas (enclosed by and including the SMA^+^ cells), automatically detecting the total number of cells by DAPI, and manually counting the SMA^+^ and/or pHH3^+^ cells using the counter tool in QuPath^54^ in three areas chosen at random. pHH3 staining of intestines and endometrium was carried out on random images taken with the Nikon Ti/E inverted microscope. QuPath was used to select SMA^−^ epithelial cells in each image, automatically detect nuclei within the selection, and automatically count the number of pHH3^+^ cells based on a threshold determined by examining positive and negative cells in the set of pictures. ER/PR status of tumors in both rat models was determined qualitatively by examining the whole slide scans. clCASP3 quantification was performed by manually selecting all the epithelium areas (enclosed by and including SMA^+^ cells) within the scanned slide, automatically detecting and counting cells in the epithelium with the DAPI stain, and manually counting the clCASP3^+^ cells in the epithelium using QuPath. CD163 quantification was performed by manually selecting all the epithelium areas (enclosed by and including SMA^+^ cells), determining the stroma and epithelium area, and manually counting the total and epithelium-associated CD163^+^ cells in three areas chosen at random.

Immunofluorescence staining for Ki67 in 3D cultures was performed, as previously described^55^. Because 3D matrigel structure is disrupted at low temperatures, all the processes were performed at room temperature. Briefly, after fixation in 2% formalin, organoids were permeabilized with 0.5% Triton X-100, blocked with blocking buffer, and stained with Ki67 antibody for 12 h. Then, after washing with IF buffer (130 mM NaCl; 7 mM Na_2_HPO_4_; 3.5 mM NaH_2_PO_4_; 7.7 mM NaN_3_; 0.1% BSA; 0.2% Triton X-100; 0.05% Tween-20), the corresponding secondary antibodies were added and incubated for 1 h. Finally, after rinsing with PBS, the slides were mounted in the Vectashield Hard Set Mounting Medium (Vector Laboratories, H1500). The images were captured by Zeiss 980 Confocal. For immunofluorescence staining of ID3, S100A4, EPCAM, KRT17, pSMAD3(Ser423/425) and pS6(Ser235/236), organoids in the 3D Matrigel structures were embedded in Histogel (Epredia, HG4000012) to make blocks. Then the blocks were sectioned and stained following the FFPE protocol described above.

### Fluorescence-activated cell sorting and flow cytometric analysis

Mammary glands were harvested and dissociated as described above and were frozen or used fresh. Single-cell suspensions were obtained by digesting with trypsin (Life Technologies, # 25300-120) at 37 °C for 5 min, followed by filtering with 100 µm strainer. Single-cell suspensions were stained with Live/Dead aqua stain (Fisher Scientific, #L34966, 1:1,000 in PBS, for 30 min at 4 °C) followed by staining for the following markers: CD24, CD29, CD31, and CD45 (Supplementary Table 1) in PBE (PBS with 0.5% BSA and 2 mM EDTA, for 30 min at 4 °C). Tumors were harvested and digested to obtain single-cell suspensions as previously described^56^. Single-cell suspensions were stained with Live/Dead aqua stain (1:1000 in PBS, for 30 min at 4 °C) followed by staining for CD45 and EPCAM (Supplementary Table 1) in PBE. Cells were analyzed and sorted using BD FACSAria II SORP UV (Becton Dickinson). Bone marrow single-cell suspensions were stained as described above for markers CD45, TCR α/β, CD3, CD11b/c, CD45ra, CD90, CD106, CD34 (Supplementary Table 1). Cells were analyzed using BD Fortessa (Becton Dickinson). All data analyses were done using FlowJo 10.6.2 (Becton Dickinson & Company).

### Serum hormone measurements

Blood was collected by decapitation in purple-capped K_2_EDTA-coated tubes (BD Microtainer#365974) and mixed immediately to prevent blood clots from forming. The samples were then centrifuged at 2000 RPM for 7 min at 4 °C. The supernatant (plasma) was transferred to another Eppendorf tube and stored at −80 °C. Serum ELISA (enzyme-linked immunosorbent assay) was performed using diluted and undiluted serum according to the manufacturer instructions for estradiol (Cayman Chemical #582251) and progesterone (Cayman Chemical # 582601) ELISA kits. Absorbances were measured using Infinite 200Pro instrument and the software Tecan i-control 1.10.4.0. The raw absorbance values were fit with a Sigmoidal curve calculated with the provided standards using Graphpad Prism software v8.

### Bulk RNA-seq analysis

Total RNA was extracted using the RNeasy Mini Kit (Qiagen). The total RNA was measured using the Agilent 2100 Bioanalyzer. RNA-seq libraries were prepared using Clontech Low Input mRNA Library (Clontech SMARTer) v4 kit from less than 10 ng of purified total RNA according to the manufacturer’s protocol. The concentrations of the finished dsDNA library were measured using the Qubit Fluorometer, the size of the library fragment was measured by Agilent TapeStation 2200, and RT-qPCR for adapted library molar concentration was measured according to the manufacturer’s protocols. Uniquely indexed libraries were pooled in equimolar ratios and sequenced on an Illumina NextSeq500 with single or paired-end reads by the Dana-Farber Cancer Institute Molecular Biology Core Facilities. RNA-seq datasets were aligned to the rat reference genome rn6 using the STAR RNA-Seq aligner (version STAR_2.5.1b) as described previously^57^. Two-pass mapping was performed using the following parameters: outSAMstrandField: intronMotif, outFilterMultimapNmax: 20, alignSJoverhangMin: 8, alignSJDBoverhangMin: 1, outFilterMismatchNmax: 999, outFilterMismatchNoverLmax: 0.1, alignIntronMin: 20, alignIntronMax: 1,000,000, alignMatesGapMax: 1,000,000, outFilterType: BySJout, outFilterScoreMinOverLread: 0.33, outFilterMatchNminOverLread: 0.33, limitSjdbInsertNsj: 1,200,000, chimSegmentMin: 15, chimJunctionOverhangMin: 15, twopassMode: Basic. Read counts for individual genes were generated using the htseq-count script of the HTSeq framework (version 0.6.1p1)^58^ using modified parameters (stranded: no) and the rn6 refGene annotation file available at the UCSC Genome Browser. Genes were filtered to retain only those with at least ten counts across all samples, then differentially expressed genes were identified by using DESeq2^59^ version 1.30.1 with a cutoff of *P*_adj_ ≤ 0.05. PCA plots and heatmaps were generated using R. Heatmaps of CD45^+^ sorted samples were done on the mouse version of the LM22 CIBERSORT signature^21^, obtained from the CIBERSORT authors, with genes converted from mouse to rat. CIBERSORT^21^ analysis was done using the web version and the converted signature, with DESeq2 normalized counts of all expressed genes as mixture files, and with the following parameters: permutations:100, quantile normalization: disabled.

### Single-cell RNA-sequencing analysis

FACS-enriched basal and luminal fractions from mammary glands from three vehicle and three and six TGFBRi-treated ACI and SD rats, respectively, were used for scRNA-seq analysis. Cell and library preparations for scRNA-seq were performed according to the Chromium Single Cell 3ʹ Reagent Kits v3 protocol (10x Genomics), targeting 5000 cells per sample. The resulting libraries were sequenced on an Illumina NextSeq500 instrument. Sequenced reads were aligned to the rat reference genome rn6 using 10x Genomics Cell Ranger 4.0.0. Total mammary gland scRNA-seq libraries from three vehicle and six TGFBRi-treated SD rats were prepared according to the Chromium Next GEM Single Cell 5’ HT v2 protocol (10x Genomics), targeting 10,000 cells per sample. Libraries were sequenced on Illumina NovaSeq S4-300 (PE150). Sequenced reads were aligned to the rat reference genome rn6 using 10x Genomics Cell Ranger 7.0.0, with the option include-introns set to off. The filtered reads assigned to cell barcodes were analyzed with the R package *Seurat*^30^, version 4.1.1 (21,763 cells for sorted ACI epithelium; 68,492 cells for sorted SD epithelium, and 111,567 cells for total SD mammary gland). In an initial quality control step, only genes expressed in at least 10 cells and only cells expressing at least 100 genes were retained. In a second step, cells with more than 15% mitochondrial genes were removed. For filtering potential contamination in the sorted epithelial data only, the quality control routine included a third step: cells with either (i) any read aligned to PTPRC (CD45^+^) or (ii) less than 2 reads aligned to any cytokeratin were removed. For the analyses and plots done on the keratin-unfiltered epithelial data presented in the manuscript, only the first two filtering steps were performed. For all the other analyses of the epithelial data, all three steps were included. The third step was omitted for the whole mammary gland data (as multiple cell types were expected to be identified), where, after quality control, a total of 103,524 cells were analyzed (40,828 control and 62,696 treated), and assigned to cell types following expression of representative markers, as follows: 28,623 epithelial cells (9891 basal, 11,242 luminal progenitor, 7490 alveolar); 46,061 immune cells (8527 T cells, 1975 NK cells, 4547 NK cells or T cells, 23,917 B cells, 4532 macrophages, 2563 monocyte-macrophages;); 27,480 stroma cells (6,094 vascular smooth muscle cells, 5442 fibroblasts, 2776 FAP + fibroblasts, 4524 endothelium, 6129 TIE1 + endothelium, 2794 stroma-progenitor, 279 vein cells), and 1081 nerve-myelin cells. The data were normalized separately for each rat strain using SCTransform^30^ and decomposed using PCA. UMAP^60^ embeddings were then computed using the first 30 PCA dimensions as input. In the sorted and filtered epithelial data, following clustering and dimensionality reduction, the two main sorted cell types (basal and luminal) were well segregated in the UMAP space. For this reason, the few cells of a sorted cell type that were part of a cluster that was at least 95% the other sorted cell type were considered mislabeled by sorting and were removed (85 basal and 32 luminal cells for ACI, and 349 basal and 31 luminal cells for SD). This amounted to retaining 16,762 ACI cells (9835 basal: 5479 control and 4356 treated; 48 secretory basal: 11 control and 37 drug; 3306 luminal progenitors: 1474 control and 1832 treated; and 3573 mature luminal: 1517 control and 2056 treated) and 39,766 SD cells (15,142 basal: 3172 control and 11,970 treated; 849 secretory: 68 control and 781 treated; 15,951 luminal progenitors: 4528 control and 11,423 treated; 7824 mature luminal: 1737 control and 6087 treated). For identifying cell subtypes separately within vehicle or treated cells, all cells from each different rat (including sorted basal and luminal) were integrated per animal using canonical correlation analysis as implemented in *Seurat*^30^. Cell subtype clusters were identified by examining the expression of representative cellular markers, as well as of top differentially expressed genes characterizing each cluster. Differential expression was done with the function *FindMarkers* in Seurat using the Wilcoxon rank-sum test (default). Lists of differentially expressed genes were generally filtered for corrected *P* value (*P*_adj_ ≤ 0.1), and further sorted decreasingly by absolute log-fold change (LFC).

The SBC signature (Supplementary Data 7) consists of 336 genes and was generated by considering positive differentially expressed genes (*P*_adj_ ≤ 0.1, LFC > 0) between the secretory basal cells and the remainder basal cells, common to both ACI and SD strains (in the sorted epithelial data). The expression of the signature in a cell was quantified by summing the normalized expression of each of its gene members in that cell.

Pseudotime analysis was performed with the R package *monocle3*^61^ version 0.2.3.0, only for the SD strain, separately for control and treated animals, including all sorted epithelial cells and integrated per animal, as discussed above. A PCA lower dimensional space was computed with the function *preprocess_cds*, without any further normalization (norm: none). The data were then visualized using UMAP. For the treated population, the starting point of the pseudotime trajectory was set to be the SBC population, while in the control cells, there were too few SBCs to be chosen as starting point. Instead, the control pseudotime trajectory was hypothesized to start from *Tp63*^+^ basal cells based on prior data demonstrating the essentiality of *Tp63* for mammary gland development^62^. Diffusion maps were inferred with the R package *destiny*^63^ version 3.4.0, with the same input as the monocle3 pseudotime analysis, using 50 diffusion components (DCs) as input.

Interactome analysis was run within the framework and R package *liana*^64^ version 0.0.1. liana aggregates several tools investigating cell–cell communication, including different algorithms and database resources. Within this framework, we ran the *Connectome*^65^ algorithm on the OmniPath database^66^ (via the R client OmniPathR), which is a composite curated resource combining several existing databases for cell–cell communication. This analysis was run only on cells from the SD strain, on the animal-level integrated data with all sorted epithelial cells included, separately for treated and control animals. The *Connectome* output data was filtered by retaining only the interactome links with corresponding *P* values <0.1 for both ligand and receptor, amounting to 199,942 inferred connections for cells belonging to the treated animals, and 63,926 connections for cells belonging to the controls. Each connection was characterized by a source and a target cluster, a ligand and a receptor, together with corresponding *P* values, and two inferred edge weights: *weight_norm*, a measure of the normalized expression of the ligand and the receptor, and *weight_sc*, a z-score scaled version of the previous metric. The pathways which were assessed for connectivity were downloaded from Panther as UNIPROT IDs using the R package *PANTHER.db*^67^ version 1.0.10, and further transformed to gene symbols using the *uniprot.org* website. In few cases, essential genes that were missing from certain pathways were added manually (colored in red in the provided supplementary table, Supplementary Data 8). Given a pathway, ligands and receptors from all the inferred connections were filtered to only members of that pathway, and additionally only connections with both edge weights positive were retained. For every cluster, the connections for which the cluster was a source were labeled as *source* or *OUT*, and the connections for which the cluster was a target as *target* or *IN*. For the cluster-level pathway plots, the number of IN and OUT connections was counted for each cluster, and further normalized to the maximum number of such connections across clusters per pathway, such that the resulting connectivity metric was a number between 0 and 1. The cell-level connectivity metric per cell type per pathway was computed by summing all IN or OUT connections belonging to clusters assigned to each cell type, and further normalizing by the total number of cells in that cell type. The cluster-level connectivity metrics in the insulin-IGF pathway plots represent the average strength of interaction, computed as the mean of either *weight_norm* or *weight_sc*, across all connections between any pair of clusters.

### Preprocessing of other single-cell RNA-sequencing datasets

The expression of the SBC signature was assessed in a recently-published scRNA-seq expression atlas of the human mammary gland^31^, which also included cells from BRCA1 mutation carriers. For the study including profiled BRCA1^+^ cells, the preprocessed data, as plotted in Fig. 4c of the original publication, were obtained from the authors upon request (59,766 cells in total, including both epithelial and non-epithelial). The epithelial cells (27,892 cells) were separated from the rest, following the authors’ assignment in the tSNE plot in Fig. 4c of the original publication. To eliminate potential remaining infiltrating stromal cells, the data was further filtered by applying a similar filter to what had been applied on our sorted epithelial rat data, namely cells with less than 2 reads aligned to any cytokeratin were removed (297 cells). The remaining 27,535 epithelial cells (12,388 from BRCA1 mutation carriers and 15,147 from non-carriers) were preprocessed with scTransform and *Seurat*^30^ as described above, and the default clustering resolution of 0.8 in *FindClusters* in *Seurat* was used for cluster assignment to epithelial cell subtypes.

The SBC signature was also assessed in a published murine mammary gland atlas^28^. The normalized scRNA-seq dataset^28^ was obtained from the authors upon request, including the subcluster labels as indicated in the original publication. In both datasets, the value of the SBC signature in each cell was computed by summing the normalized expression of all SBC genes also present in the dataset, mapped to their respective homologs.

### Additional bioinformatic analyses

Pathway enrichment analysis was performed using MetaCore v20.4 (https://portal.genego.com), on differentially expressed genes from bulk and scRNA-seq analyses (genes with *P*_adj_ ≤ 0.05 for bulk RNA-seq; *P*_adj_ ≤ 0.05, absolute LFC ≥ 0.5 for scRNA-seq). Gene lists were analyzed for enriched process networks using as cutoffs for significant enrichment *P* value <0.001 and *P*_adj_ ≤ 0.05. Gene lists were also subjected to protein network analysis using STRING v11.0 (https://string-db.org). Enrichments from Gene Ontology (biological process), KEGG, and Reactome pathways from the STRING analysis were used to group proteins with similar molecular function and the resulting grouping was further manually curated using a literature search.

Pre-ranked GSEA analysis^68^ was run to assess the enrichment of the SBC signature on: (i) differentially expressed genes derived from Serial Analysis of Gene Expression (SAGE) data of CD10-CD24-CD44 + normal breast tissue of different phenotypes (nulliparous vs. parous women, *BRCA1/2* mutation carriers vs. non-carriers), filtered by *P*_adj_ ≤ 0.1 and ranked by fold change, as reported in our previous work^6^, (ii) pre-defined Chemical and genomic perturbations (CGP) GSEA pathways.

### Reporting summary

Further information on research design is available in the Nature Portfolio Reporting Summary linked to this article.

## Supplementary information

Supplementary Information

Reporting Summary

Description of Additional Supplementary Files

Supplementary Dataset 1

Supplementary Dataset 2

Supplementary Dataset 3

Supplementary Dataset 4

Supplementary Dataset 5

Supplementary Dataset 6

Supplementary Dataset 7

Supplementary Dataset 8

## Data Availability

The human^31^ and mouse^28^ publicly available single-cell RNA-seq data used in this study are available in the GEO database under accession numbers GSE106273 and GSE161529, respectively. The RNA-seq and scRNA-seq data generated in this study have been deposited in the NCBI GEO database under accession number GSE184095. The preprocessed genomics data objects generated and used here have been deposited in Zenodo, 10.5281/zenodo.7293642 (Zenodo link https://zenodo.org/record/7293642#.Y41G9i-B19f). The remaining data are available within the Article, Supplementary Information, or Source Data file. Source data are provided with this paper.

## Source data

source data

## Code availability

All code used to analyze genomics data and produce the corresponding figures is available on the GitHub repository https://github.com/csimona/tumor-prevention-rat-scRNAseq.
