# Supplementary material for: Breast cancer prevention by short-term inhibition of TGFβ signaling
Source: Nat Commun. 2022 Dec 7;13:7558. doi: 10.1038/s41467-022-35043-5 (PMC9729304; doi:10.1038/s41467-022-35043-5)
Supplement: Supplementary file 1 — Supplementary Information [file 41467_2022_35043_MOESM1_ESM.pdf]

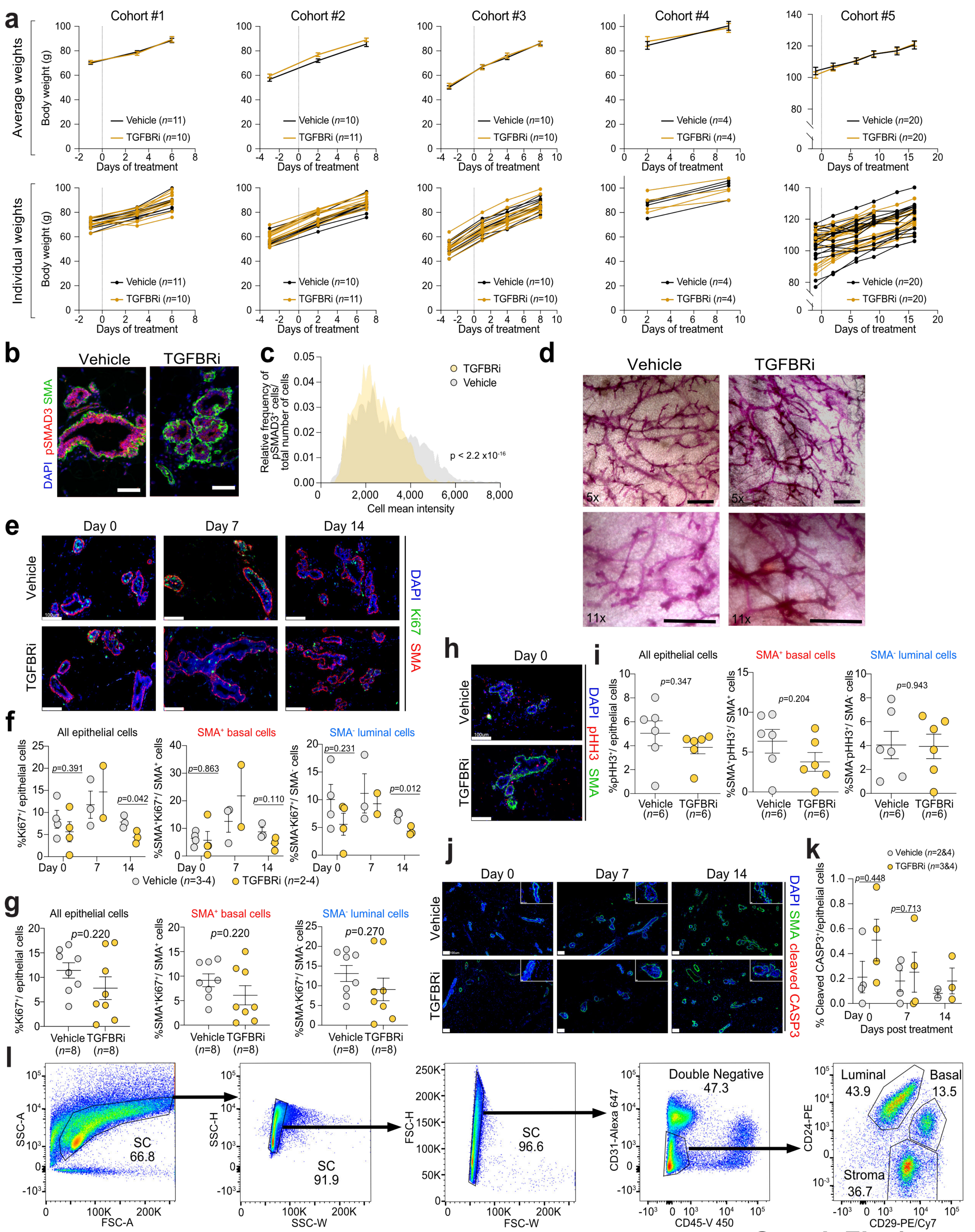

a
Cohort #1
Cohort #2
Cohort #3
Cohort #4
Cohort #5
Average weights
Individual weights
Body weight (g)
Days of treatment
Vehicle (n=11)
TGFBRi (n=10)
Vehicle (n=10)
TGFBRi (n=11)
Vehicle (n=10)
TGFBRi (n=10)
Vehicle (n=4)
TGFBRi (n=4)
Vehicle (n=20)
TGFBRi (n=20)
b
Vehicle
TGFBRi
DAPI pSMAD3 SMA
c
TGFBRi
Vehicle
Relative frequency of pSMAD3+ cells/ total number of cells
p < 2.2 x10-16
Cell mean intensity
d
Vehicle
TGFBRi
5x
11x
e
Day 0
Day 7
Day 14
Vehicle
TGFBRi
DAPI Ki67 SMA
f
All epithelial cells
SMA+ basal cells
SMA- luminal cells
%Ki67+/ epithelial cells
%SMA+Ki67+/ SMA+ cells
%SMA-Ki67+/ SMA- cells
p=0.391
p=0.042
p=0.863
p=0.110
p=0.231
p=0.012
Vehicle (n=3-4)
TGFBRi (n=2-4)
g
All epithelial cells
SMA+ basal cells
SMA- luminal cells
p=0.220
p=0.220
p=0.270
Vehicle (n=8)
TGFBRi (n=8)
h
Day 0
Vehicle
TGFBRi
DAPI pHH3 SMA
i
All epithelial cells
SMA+ basal cells
SMA- luminal cells
%pHH3+/ epithelial cells
%SMA+pHH3+/ SMA+ cells
%SMA-pHH3+/ SMA- cells
p=0.347
p=0.204
p=0.943
Vehicle (n=6)
TGFBRi (n=6)
j
Day 0
Day 7
Day 14
Vehicle
TGFBRi
DAPI SMA cleaved CASP3
k
Vehicle (n=2&4)
TGFBRi (n=3&4)
% Cleaved CASP3+/epithelial cells
p=0.448
p=0.713
Days post treatment
l
SSC-A
FSC-A
SC 66.8
SSC-H
SSC-W
SC 91.9
FSC-H
FSC-W
SC 96.6
CD31-Alexa 647
CD45-V 450
Double Negative 47.3
CD24-PE
CD29-PE/Cy7
Luminal 43.9
Basal 13.5
Stroma 36.7

Suppl Fig 1

Supplementary Figure 1. The effect of peripubertal TGFBRi treatment on ACI rats. a, Body weights throughout the experiment of female ACI rats treated with vehicle control or TGFBRi (LY2157299) twice daily for 10 (cohorts #1-4) or 21 (cohort #5) days. In cohorts 1 and 2 the number of rats/group varied at different days after starting treatment due to loss of animals during the course of the experiment. Measurements were started before treatment and taken during the experiment. Top graphs show the average body weights per group +/- s.e.m. Bottom graphs show the individual body weights of each animal over time. For cohort #1, vehicle n=11, TGFBRi n=10. For cohort #2, vehicle n=10, TGFBRi n=11. For cohort #3, vehicle n=10, TGFBRi n=10. For cohort #4, vehicle n=4, TGFBRi n=4. For cohort #5, vehicle n=20, TGFBRi n=20. No significance between the body weights of vehicle and TGFBRi-treated animals was found in any cohort. **b,c**, Multicolor immunofluorescence analysis (**b**) and quantification (**c**) of phosphorylated SMAD3 (pSMAD3) in vehicle and TGFBRi-treated animals. **d**, Representative images of mammary gland whole mounts to visualize TEBs (5x and 11x magnification). **e-i**, Multicolor immunofluorescence analysis (**e,h**) and quantification (**f,g,i**) of Ki67^{+} (**e-g**) and pHH3^{+} (**h,i**) cells within the indicated mammary epithelial cell populations and time points after treatment was stopped. Number of animals analyzed varied at different time points (**f**); day 0 (vehicle n=4, TGFBRi n=4), day 7 (vehicle n=3, TGFBRi n=2), and day 14 (vehicle n=3, TGFBRi n=3). **g** shows multiple cohorts at day 0. **j,k**, Representative multicolor immunofluorescence images (**j**) and quantification (**k**) of cleaved caspase 3-positive (cleaved CASP3^{+}, red) cells in the mammary epithelium of vehicle- and TGFBRi-treated ACI rats on different days after treatment was stopped. **l**, Gating strategy used for polychromatic flow cytometry analyses of mammary glands dissociated to single cells. All graphs (**f,g,i,k**) are presented as mean ± s.e.m. *P*-values indicate statistical significance of difference between vehicle and TGFBRi-treated groups and were calculated by: unpaired two-tailed *t*-test with the Holm-Sidak correction (alpha = 0.05) (**a**), Kolmogorov-Smirnov (KS) test (**c**), unpaired two-tailed *t*-test with Welch's correction (**f,g,i,k**). Scale bars: full images 100 μm (**b,e,h,j**), 1mm (**d**), inset 10 μm (**j**). Source data are provided as a Source Data file.

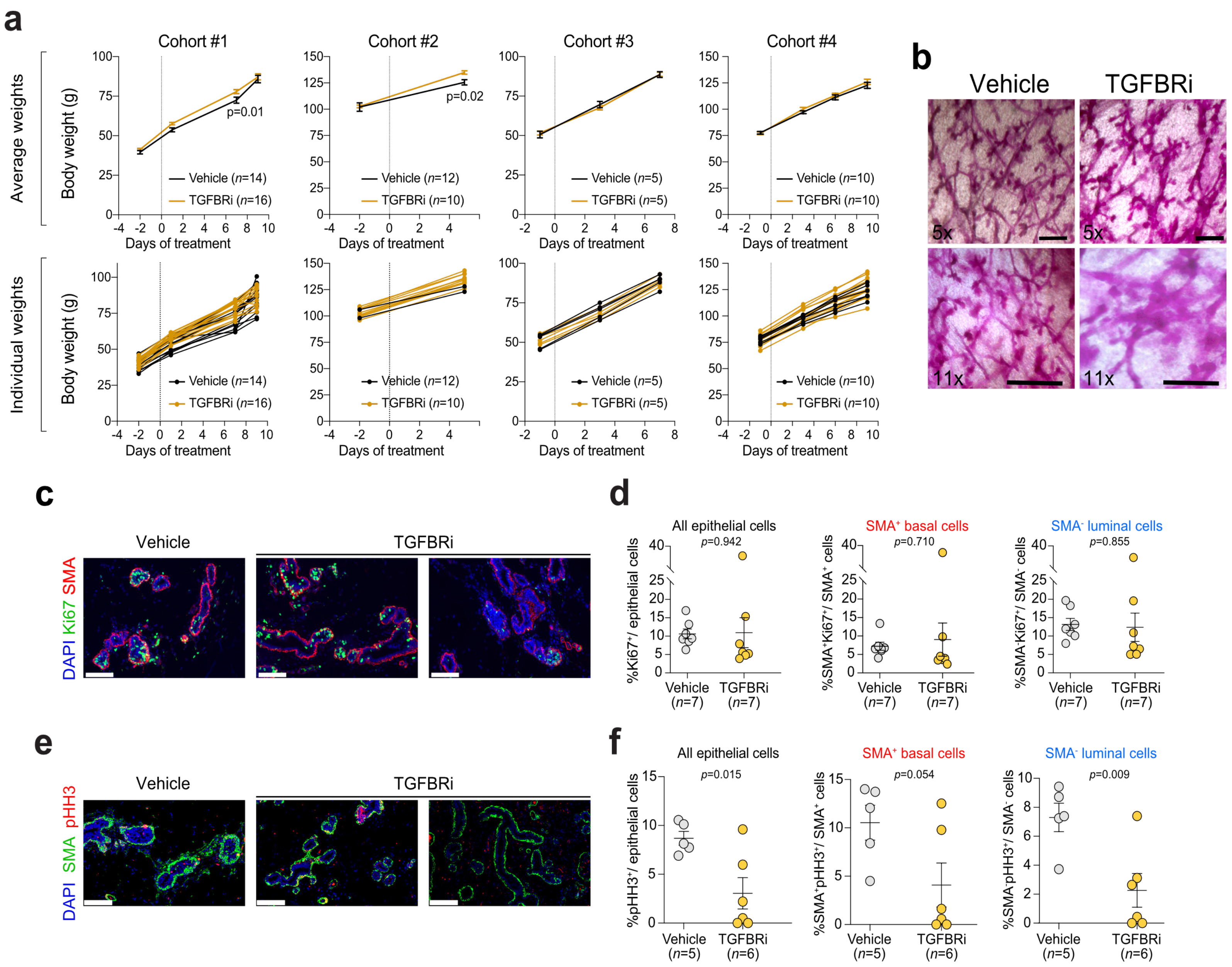
a
Cohort #1
Cohort #2
Cohort #3
Cohort #4
Average weights
Body weight (g)
p=0.01
p=0.02
Vehicle (n=14)
TGFBRi (n=16)
Vehicle (n=12)
TGFBRi (n=10)
Vehicle (n=5)
TGFBRi (n=5)
Vehicle (n=10)
TGFBRi (n=10)
Days of treatment
Individual weights
b
Vehicle
TGFBRi
5x
11x
c
Vehicle
TGFBRi
DAPI Ki67 SMA
d
All epithelial cells
SMA+ basal cells
SMA- luminal cells
p=0.942
p=0.710
p=0.855
%Ki67+/ epithelial cells
%SMA+Ki67+/ SMA+ cells
%SMA-Ki67+/ SMA- cells
Vehicle (n=7)
TGFBRi (n=7)
e
DAPI SMA pHH3
f
p=0.015
p=0.054
p=0.009
%pHH3+/ epithelial cells
%SMA+pHH3+/ SMA+ cells
%SMA-pHH3+/ SMA- cells
Vehicle (n=5)
TGFBRi (n=6)

Suppl Fig. 2

Supplementary Figure 2. The effect of peripubertal TGFBRi treatment on SD rats. a, Body weights throughout the experiment of female SD rats treated with vehicle control or TGFBRi (LY2157299) twice daily for 10 days. Measurements were started before treatment and taken during the experiment. Top graphs show the average body weights per group +/- s.e.m. Bottom graphs show the individual body weights of each animal over time. Significance was found at one time point in cohort #1 and cohort #2 (*p*-values indicated on the plots), with TGFBRi-treated rats weighing more than vehicle-treated animals. **b**, Representative images of mammary gland whole mounts to visualize TEBs (5x and 11x magnification) from multiple animals (vehicle n=3, TGFBRi n=3) analyzed. **c-f**, Representative multicolor immunofluorescence images (**c,e**) and quantification (**d,f**) of $Ki67^+$ (**c,d**) and $pHH3^+$ (**e,f**) cell populations in the mammary epithelium of vehicle- and TGFBRi-treated SD rats at day 0 after treatment was stopped. All graphs (**d,f**) are presented as mean ± s.e.m. *P*-values indicate statistical significance of difference between vehicle- and TGFBRi-treated groups and were calculated by: unpaired, two-tailed *t*-test with the Holm-Sidak correction (alpha = 0.05) (**a**); and unpaired, two-tailed *t*-test with Welch's correction (**d,f**). Scale bars 1mm (**b**), 100 µm (**c,e**). Source data are provided as a Source Data file.

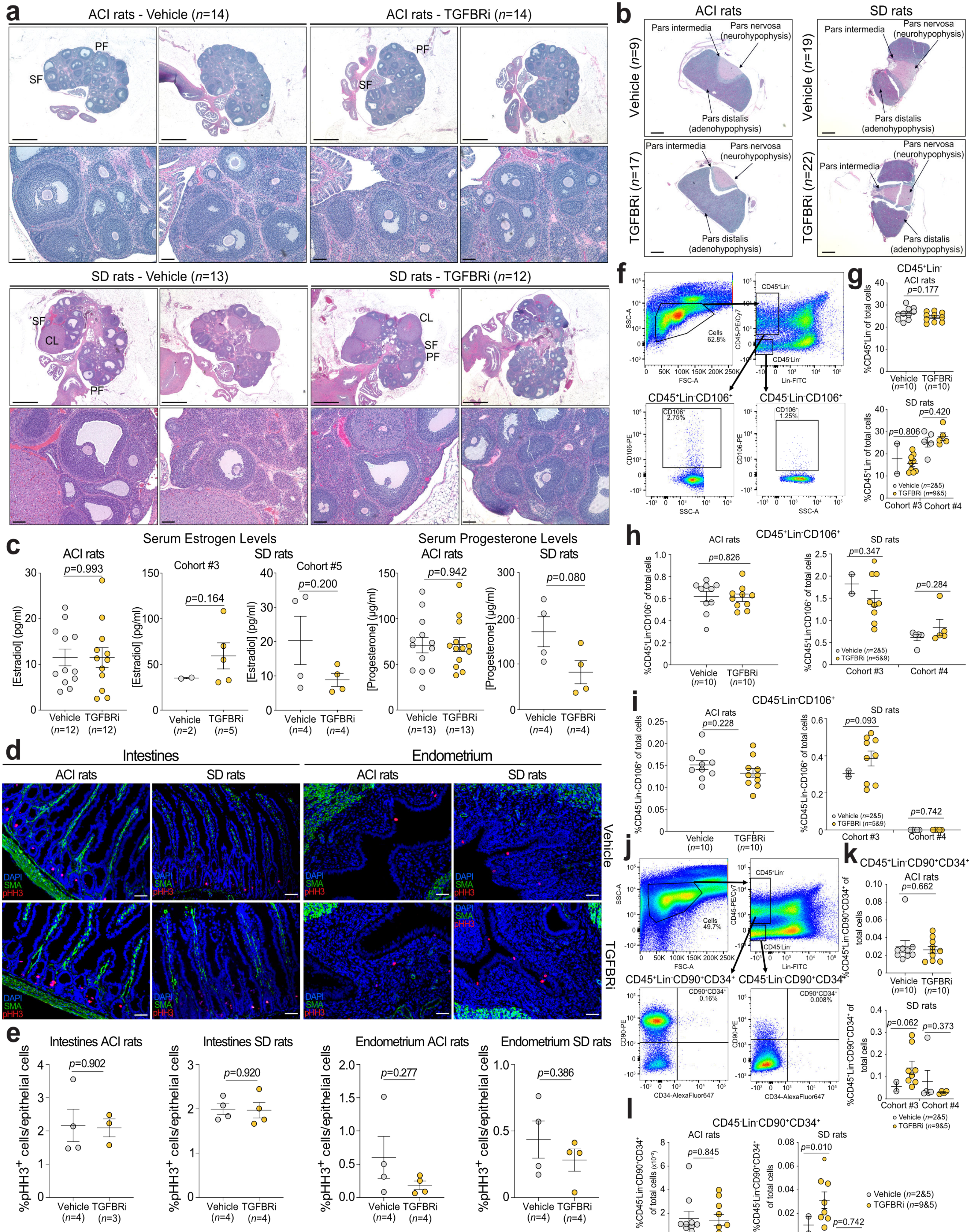

a
ACI rats - Vehicle (n=14)
ACI rats - TGFBRi (n=14)
SD rats - Vehicle (n=13)
SD rats - TGFBRi (n=12)
b
ACI rats
SD rats
Pars intermedia
Pars nervosa (neurohypophysis)
Pars distalis (adenohypophysis)
Vehicle (n=9)
TGFBRi (n=17)
Vehicle (n=19)
TGFBRi (n=22)
c
Serum Estrogen Levels
Serum Progesterone Levels
d
Intestines
Endometrium
ACI rats
SD rats
Vehicle
TGFBRi
e
Intestines ACI rats
Intestines SD rats
Endometrium ACI rats
Endometrium SD rats
f
g
CD45+Lin-
h
CD45+Lin-CD106+
i
CD45-Lin-CD106+
j
k
CD45+Lin-CD90+CD34+
l
CD45-Lin-CD90+CD34+

Suppl Fig 3

Supplementary Figure 3. The effects of peripubertal TGFBRi treatment on other organs. a, Representative H&E images of ovaries from control and TGFBRi-treated ACI (*top*) and SD (*bottom*) rats. Primary follicle (PF), secondary follicle (SF), and corpus luteum (CL) are indicated. Total number of animals analyzed in each experimental condition is indicated in parentheses. **b**, Representative H&E images of pituitary glands (hypophysis) from control and TGFBRi-treated ACI (*left)* and SD (*right*) rats. The three histologically distinct parts reflecting histology are indicated. **c**, Serum estradiol and progesterone hormone levels of control and TGFBRi-treated ACI and SD rats. **d,e**, Representative multicolor immunofluorescence images (**d**) and quantification (**e**) of $pHH3^+$ epithelial (SMA^-) cells in the intestinal tract and endometrium of control and TGFBRi-treated ACI and SD rats. Quantification represents the average fraction of $pHH3^+$ cells per animal based on three random areas. **f-l**, Flow cytometry gating strategy (**f,j**) and quantification (**g-i,k,l**) of bone marrow hematopoietic (**g,h,k**) and mesenchymal (**i,l**) progenitor/stem cells. All graphs (**c,e,g,h,i,k,l**) are presented as mean ± s.e.m. *P*-values were calculated by unpaired, two-tailed *t*-test with Welch's correction. Scale bars: 1 mm (**a**); 0.5 mm (**b**); 50 µm (**d**). Source data are provided as a Source Data file.

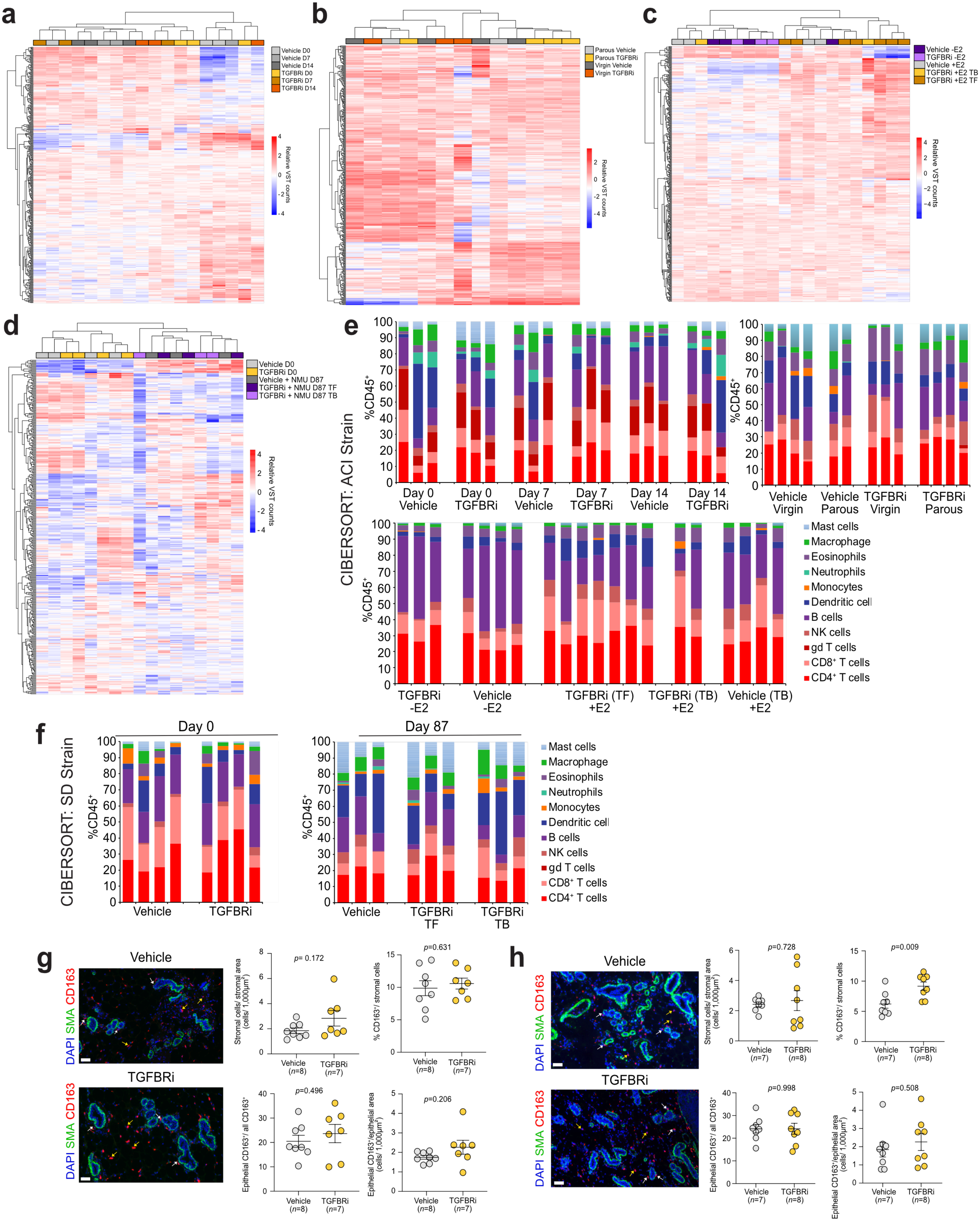

a
Vehicle D0
Vehicle D7
Vehicle D14
TGFBRi D0
TGFBRi D7
TGFBRi D14
Relative VST counts
b
Parous Vehicle
Parous TGFBRi
Virgin Vehicle
Virgin TGFBRi
c
Vehicle -E2
TGFBRi -E2
Vehicle +E2
TGFBRi +E2 TB
TGFBRi +E2 TF
d
Vehicle D0
TGFBRi D0
Vehicle + NMU D87
TGFBRi + NMU D87 TF
TGFBRi + NMU D87 TB
e
CIBERSORT: ACI Strain
%CD45+
Day 0 Vehicle
Day 0 TGFBRi
Day 7 Vehicle
Day 7 TGFBRi
Day 14 Vehicle
Day 14 TGFBRi
Vehicle Virgin
Vehicle Parous
TGFBRi Virgin
TGFBRi Parous
TGFBRi -E2
Vehicle -E2
TGFBRi (TF) +E2
TGFBRi (TB) +E2
Vehicle (TB) +E2
Mast cells
Macrophage
Eosinophils
Neutrophils
Monocytes
Dendritic cell
B cells
NK cells
gd T cells
CD8+ T cells
CD4+ T cells
f
CIBERSORT: SD Strain
Day 0
Day 87
%CD45+
Vehicle
TGFBRi
TGFBRi TF
TGFBRi TB
g
Vehicle
TGFBRi
DAPI SMA CD163
p= 0.172
p=0.631
p=0.496
p=0.206
Stromal cells/ stromal area (cells/ 1,000µm2)
% CD163+/ stromal cells
Epithelial CD163+/ all CD163+
Epithelial CD163+/epithelial area (cells/ 1,000µm2)
Vehicle (n=8)
TGFBRi (n=7)
h
p=0.728
p=0.009
p=0.998
p=0.508
Vehicle (n=7)
TGFBRi (n=8)

Suppl Fig. 4

Supplementary Figure 4. The effects of TGFBRi treatment on mammary gland leukocytes. **a-d**, Heatmaps depicting hierarchical clustering of bulk RNA-seq data of $CD45^+$ mammary gland leukocytes from ACI (**a-c**) and SD (**d**) rats based on differentially expressed immune-related genes. Leukocyte fractions were analyzed at 0 (D0), 7 (D7) and 14 (D14) days after TGFBRi treatment was ended for peripubertal ACI rats (**a**), as well as immediately after TGFBRi treatment was stopped for adult virgin or parous ACI (**b**) and peripubertal SD rats denoted in the legend with D0 (**d**). In a separate experiment, SD rats were treated with the carcinogen NMU following TGFBRi treatment and leukocyte composition was assessed 87 days later in mammary glands from vehicle-treated animals with tumors and TGFBRi-treated animals with (tumor-bearing, TB) and without (tumor-free, TF) tumors (**d**). In another separate experiment, ACI rats were treated with estradiol (+E2) to induce tumors, and leukocytes were assessed at the study endpoint in normal mammary glands from vehicle-treated animals with tumors and TGFBRi-treated animals with (tumor-bearing, TB) and without (tumor-free, TF) tumors (**c**). ACI rats that were not treated with estradiol (-E2) did not develop tumors by the end of the study. **e,f,** CIBERSORT analysis estimating the relative abundance of distinct immune cell types in RNA-seq of $CD45^+$ mammary gland leukocytes of ACI (**e**) and SD (**f**) rats at indicated experimental condition and timepoint. **g,h**, Representative multicolor immunofluorescence images (*left*) and quantification (*right*) of $CD163^+$ macrophages in mammary glands of vehicle and TGFBRi-treated ACI (**g**) and SD (**h**) rats. SMA (green) marks myoepithelial cells, DAPI (blue) marks nuclei. Arrows highlight macrophages that are in direct contact (white) or not in contact (yellow) with the mammary epithelium. All graphs (**g,h**) are presented as mean ± s.e.m. *P*-values were calculated by unpaired, two-tailed *t*-test with Welch's correction comparing treatment vs. vehicle. Scale bars: 50 μm. Source data are provided as a Source Data file.

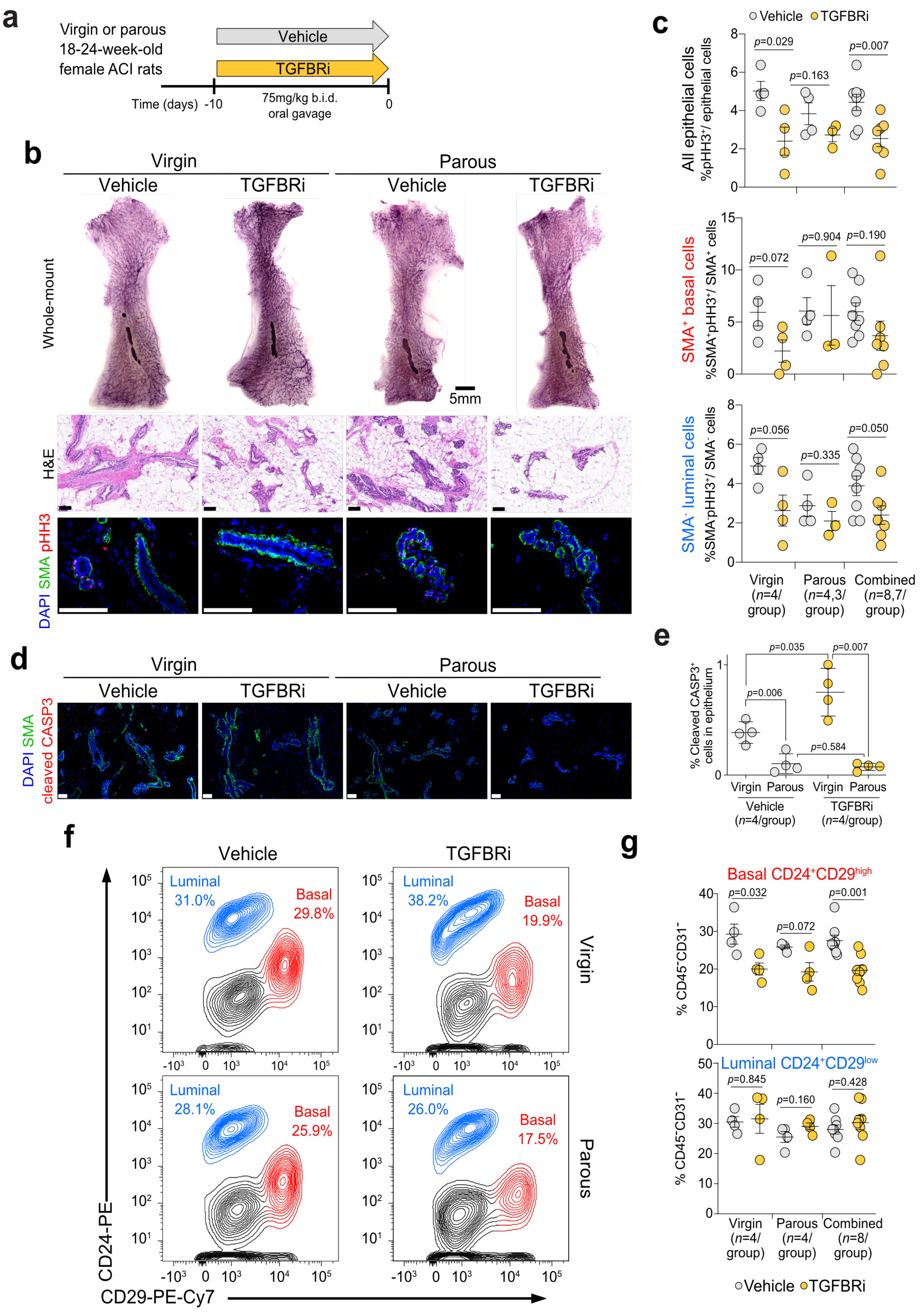
a
Virgin or parous
18-24-week-old
female ACI rats
Vehicle
TGFBRi
Time (days) -10
75mg/kg b.i.d.
oral gavage
0
b
Virgin
Parous
Vehicle
TGFBRi
Vehicle
TGFBRi
Whole-mount
5mm
H&E
DAPI SMA pHH3
c
Vehicle TGFBRi
All epithelial cells
%pHH3+/ epithelial cells
p=0.029
p=0.163
p=0.007
SMA+ basal cells
%SMA+pHH3+/ SMA+ cells
p=0.072
p=0.904
p=0.190
SMA- luminal cells
%SMA-pHH3+/ SMA- cells
p=0.056
p=0.335
p=0.050
Virgin (n=4/ group)
Parous (n=4,3/ group)
Combined (n=8,7/ group)
d
Virgin
Parous
Vehicle
TGFBRi
Vehicle
TGFBRi
DAPI SMA cleaved CASP3
e
% Cleaved CASP3+ cells in epithelium
p=0.035
p=0.007
p=0.006
p=0.584
Virgin Parous Virgin Parous
Vehicle (n=4/group)
TGFBRi (n=4/group)
f
Vehicle
TGFBRi
Luminal 31.0%
Basal 29.8%
Luminal 38.2%
Basal 19.9%
Virgin
Luminal 28.1%
Basal 25.9%
Luminal 26.0%
Basal 17.5%
Parous
CD24-PE
CD29-PE-Cy7
g
Basal CD24+CD29high
% CD45-CD31-
p=0.032
p=0.072
p=0.001
Luminal CD24+CD29low
p=0.845
p=0.160
p=0.428
Virgin (n=4/ group)
Parous (n=4/ group)
Combined (n=8/ group)
Vehicle TGFBRi

Suppl Fig. 5

Supplementary Figure 5. The effect of TGFBRi treatment in adulthood on mammary glands of ACI rats. a, Schematic outline of experimental design of vehicle or TGFBRi treatment of adult virgin and parous ACI rats. **b**, Representative mammary gland whole mounts, H&E staining, and multicolor immunofluorescence (IF) of SMA (green) and pHH3 (red). DAPI (blue) marks nuclei. Results are representative of multiple animals analyzed (vehicle n=4, TGFBRi n=4 in both virgin and parous groups). **c**, Relative frequencies of $pHH3^+$ cells in the indicated cell populations. **d,e**, Representative multicolor IF images (**d**) and quantification (**e**) of the frequencies of cleaved $CASP3^+$ cells in indicated cell types and treatment regimens of mammary glands stained with SMA (green), cleaved CASP3 (red), and DAPI (blue). **f,g**, Representative flow cytometry plots (**f**) and quantification (**g**) of basal ($CD24^+CD29^{high}$) and luminal ($CD24^+CD29^{low}$) mammary epithelial cells within the $CD31^-CD45^-$ cell populations. Graphs are presented as mean ± s.e.m. *P*-values were calculated by unpaired two-tailed *t*-test with Welch's correction. Scale bars: whole mounts 5 mm, H&E and IF images: 100 µm. "Combined" indicates pooled data from all virgin and parous rats. Source data are provided as a Source Data file.

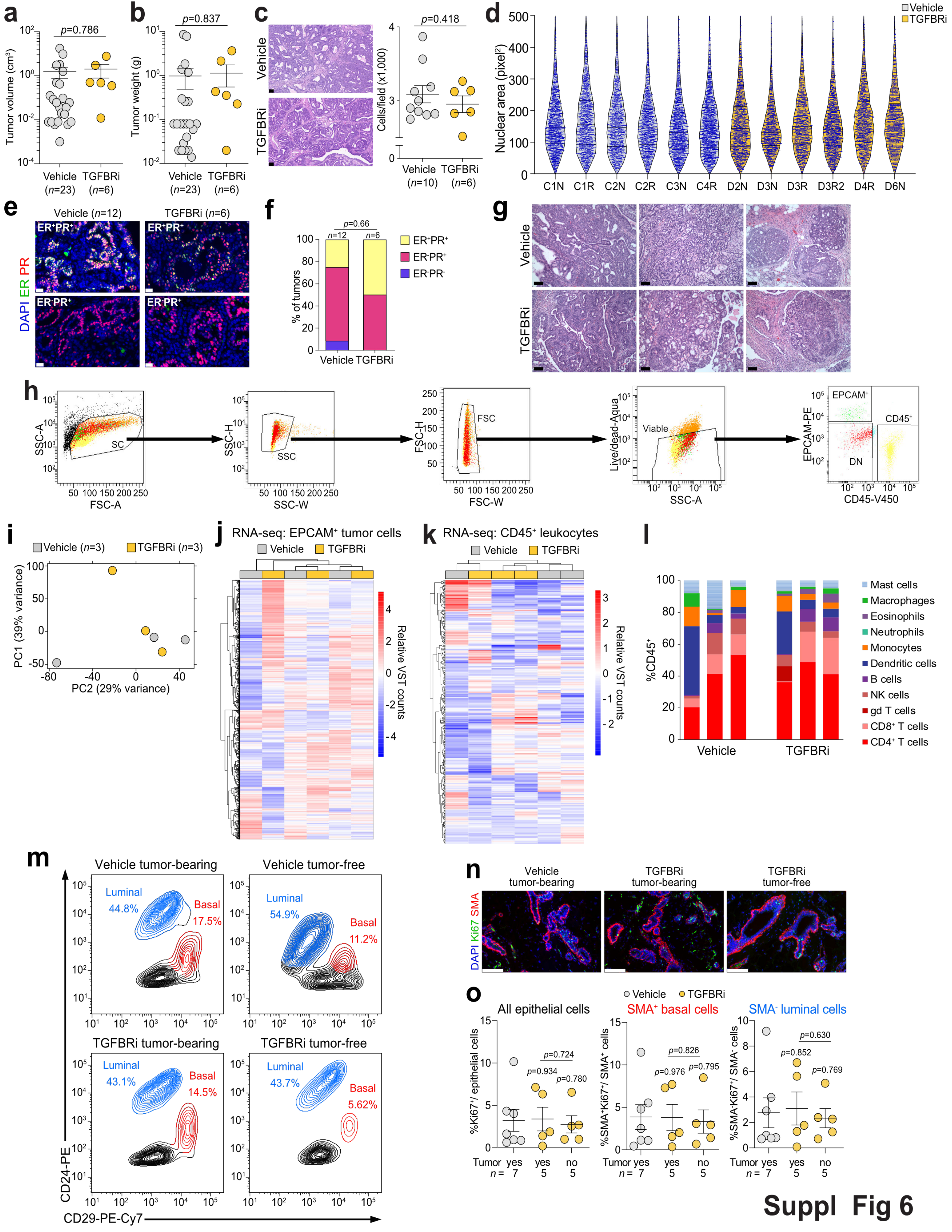

a
p=0.786
Tumor volume (cm³)
Vehicle (n=23)
TGFBRi (n=6)
b
p=0.837
Tumor weight (g)
Vehicle (n=23)
TGFBRi (n=6)
c
Vehicle
TGFBRi
p=0.418
Cells/field (x1,000)
Vehicle (n=10)
TGFBRi (n=6)
d
Vehicle
TGFBRi
Nuclear area (pixel²)
C1N C1R C2N C2R C3N C4R D2N D3N D3R D3R2 D4R D6N
e
Vehicle (n=12)
TGFBRi (n=6)
DAPI ER PR
ER⁺PR⁺
ER⁻PR⁺
f
p=0.66
n=12
n=6
% of tumors
ER⁺PR⁺
ER⁻PR⁺
ER⁻PR⁻
Vehicle TGFBRi
g
Vehicle
TGFBRi
h
SSC-A
FSC-A
SC
SSC-H
SSC-W
SSC
FSC-H
FSC-W
FSC
Live/dead-Aqua
SSC-A
Viable
EPCAM-PE
CD45-V450
EPCAM⁺
CD45⁺
DN
i
Vehicle (n=3)
TGFBRi (n=3)
PC1 (39% variance)
PC2 (29% variance)
j
RNA-seq: EPCAM⁺ tumor cells
Vehicle
TGFBRi
Relative VST counts
k
RNA-seq: CD45⁺ leukocytes
Vehicle
TGFBRi
Relative VST counts
l
%CD45⁺
Mast cells
Macrophages
Eosinophils
Neutrophils
Monocytes
Dendritic cells
B cells
NK cells
gd T cells
CD8⁺ T cells
CD4⁺ T cells
Vehicle
TGFBRi
m
Vehicle tumor-bearing
Luminal 44.8%
Basal 17.5%
Vehicle tumor-free
Luminal 54.9%
Basal 11.2%
TGFBRi tumor-bearing
Luminal 43.1%
Basal 14.5%
TGFBRi tumor-free
Luminal 43.7%
Basal 5.62%
CD24-PE
CD29-PE-Cy7
n
Vehicle tumor-bearing
TGFBRi tumor-bearing
TGFBRi tumor-free
DAPI Ki67 SMA
o
Vehicle
TGFBRi
All epithelial cells
%Ki67⁺/ epithelial cells
p=0.724
p=0.934
p=0.780
SMA⁺ basal cells
%SMA⁺Ki67⁺/ SMA⁺ cells
p=0.826
p=0.976
p=0.795
SMA⁻ luminal cells
%SMA⁻Ki67⁺/ SMA⁻ cells
p=0.630
p=0.852
p=0.769
Tumor yes yes no
n = 7 5 5

Suppl Fig 6

Supplementary Figure 6. Characterization of NMU-induced tumors in SD rats. a,b, Tumor volume (**a**) and weight (**b**) of NMU-induced mammary tumors in SD rats. **c,** Representative H&E-stained images and quantification of tumor cellularity. **d**, Quantification of tumor cell nuclear area. Each violin plot depicts the data obtained from three random areas (each dot is a data point) from one individual tumor (indexed on the horizontal axis). **e,f**, Representative images (**e**) and quantification (**f**) of estrogen receptor (ER) and progesterone receptor (PR) expression in tumors. **g**, Representative H&E-stained images of different areas of NMU-induced mammary tumors in vehicle and TGFBRi-treated SD rats highlighting topological and cellular heterogeneity. All tumors in both vehicle (n=9) and TGFBRi (n=5) treated groups showed the same histology. **h**, FACS gating strategy used to purify tumor cells (EPCAM$^+$) and leukocytes (CD45$^+$) for RNA-seq from NMU-induced mammary tumors dissociated into single cells. **i** PCA plot of EPCAM$^+$ tumor RNA-seq data from vehicle and TGFBRi-treated SD rats who developed tumors. **j**, Heatmap of RNA-seq EPCAM$^+$ data from NMU-induced mammary gland tumors from vehicle- and TGFBRi-treated SD rats, clustered based on the top 10% most differentially expressed genes among the two conditions. **k**, Heatmap of RNA-seq CD45$^+$ leukocytes data from NMU-induced mammary gland tumors from vehicle- and TGFBRi-treated SD rats, based on differentially expressed immune-related genes. **l**, CIBERSORT analysis estimating the relative abundance of distinct immune cell types in RNA-seq data of CD45$^+$ leukocytes from NMU-induced mammary gland tumors from vehicle- and TGFBRi-treated SD rats (3 replicates per condition). **m**, Representative flow cytometric analysis of luminal (CD24$^+$CD29^{low}) and basal (CD24$^+$CD29^{high}) mammary epithelial cells within the CD31$^-$CD45$^-$ cell population. **n,o,** Representative immunofluorescence images (**n**) and quantification of SMA$^+$ and Ki67$^+$ cells (**o**) in SD rat mammary glands. TGFBRi-treated tumor-bearing (yes) and tumor-free (no) rats were compared to vehicle-treated ones as well as to each other. DAPI marks nuclei. Graphs are presented as mean ± s.e.m. *P*-values were calculated by unpaired, two-tailed *t*-test with Welch's correction (**a-c,o**); two-sided Fisher's exact test (**f**). Scale bars: 100 μm (**c,n**), 20 μm (**e**), 200 μm (**g**). Source data are provided as a Source Data file.

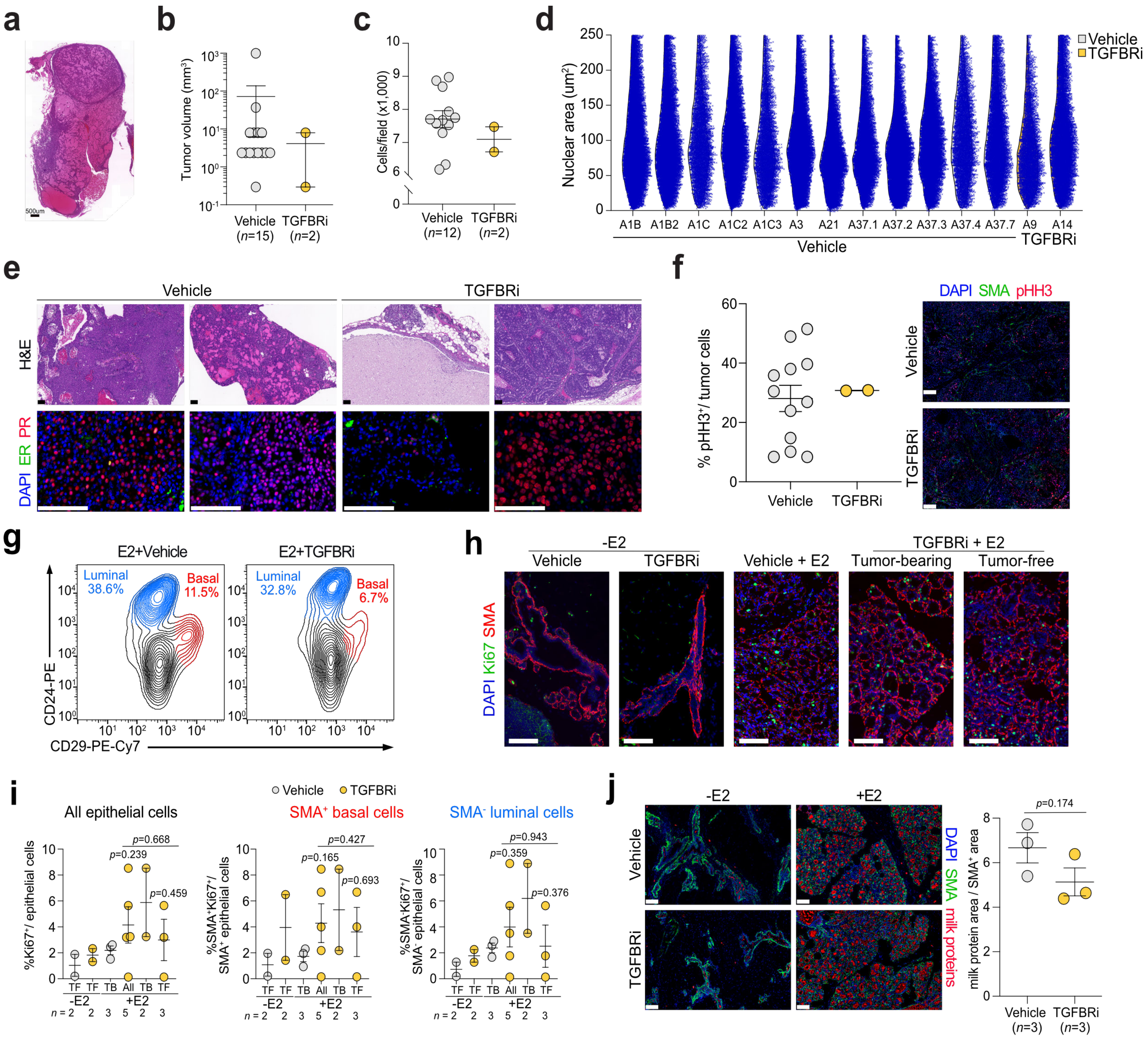
a
500um
b
Tumor volume (mm³)
Vehicle (n=15)
TGFBRi (n=2)
c
Cells/field (x1,000)
Vehicle (n=12)
TGFBRi (n=2)
d
Nuclear area (um²)
Vehicle
TGFBRi
A1B A1B2 A1C A1C2 A1C3 A3 A21 A37.1 A37.2 A37.3 A37.4 A37.7 A9 A14
e
Vehicle
TGFBRi
H&E
DAPI ER PR
f
% pHH3⁺/ tumor cells
DAPI SMA pHH3
g
E2+Vehicle
E2+TGFBRi
Luminal 38.6%
Basal 11.5%
Luminal 32.8%
Basal 6.7%
CD24-PE
CD29-PE-Cy7
h
-E2
Vehicle
TGFBRi
Vehicle + E2
TGFBRi + E2
Tumor-bearing
Tumor-free
DAPI Ki67 SMA
i
All epithelial cells
SMA⁺ basal cells
SMA⁻ luminal cells
%Ki67⁺/ epithelial cells
%SMA⁺Ki67⁺/ SMA⁺ epithelial cells
%SMA⁻Ki67⁺/ SMA⁻ epithelial cells
p=0.668
p=0.239
p=0.459
p=0.427
p=0.165
p=0.693
p=0.943
p=0.359
p=0.376
TF TF TB All TB TF
-E2 +E2
n = 2 2 3 5 2 3
j
-E2
+E2
DAPI SMA milk proteins
milk protein area / SMA⁺ area
p=0.174
Vehicle (n=3)
TGFBRi (n=3)

Suppl Fig 7

Supplementary Figure 7. Characterization of E2-induced tumors in ACI rats. a, Representative image of a pituitary adenoma in E2-treated ACI rats. **b-d**, Quantification of tumor volume (**b**), tumor cellularity (**c**) and nuclear area (**d**) of E2-induced mammary tumors in ACI rats. Each violin plot depicts the data obtained from three random areas (each dot is a data point) from one individual tumor. **e**, Representative H&E-stained sections (*top*) and immunofluorescence (IF) analysis (*bottom*) of estrogen receptor (ER, green) and progesterone receptor (PR, red) of mammary gland tumors from E2-implanted ACI animals treated with vehicle or TGFBRi, illustrating the tumor heterogeneity in the tumor model. Analysis of multiple tumors from both E2+vehicle (n=6) and E2+TGFBRi (n=2) groups gave similar results. **f**, Relative fraction of $pHH3^+$ tumor cells in E2-induced mammary tumors in ACI rats (*left*) and representative immunofluorescence images (*right*). Immunofluorescence was performed once on multiple samples from vehicle (n=12) and TGFBRi (n=2) treated rats. **g**, Representative flow cytometric analysis of luminal ($CD24^+CD29^{low}$) and basal ($CD24^+CD29^{high}$) mammary epithelial cells within the $CD31^-CD45^-$ cell population. **h,i**, Representative immunofluorescence images (**h**) and quantification (**i)** of $Ki67^+$ across SMA^+ and SMA^- mammary epithelial cells. P values indicate significance of differences between TGFBRi-treated E2-treated tumor bearing (TB) and tumor-free (TF) animals. **j**, Representative images of IF staining for milk protein (red) in mammary glands of untreated (-E2) and estradiol-treated (+E2) ACI rats (*left*). Milk protein area relative to SMA^+ area was quantified for +E2 animals (*right*). Graphs are presented as mean ± s.e.m. *P*-values were calculated by unpaired, two-tailed *t*-test with Welch's correction. Scale bars: 500 µm (**a**), 100 µm (**e,f,h,j**). Source data are provided as a Source Data file.

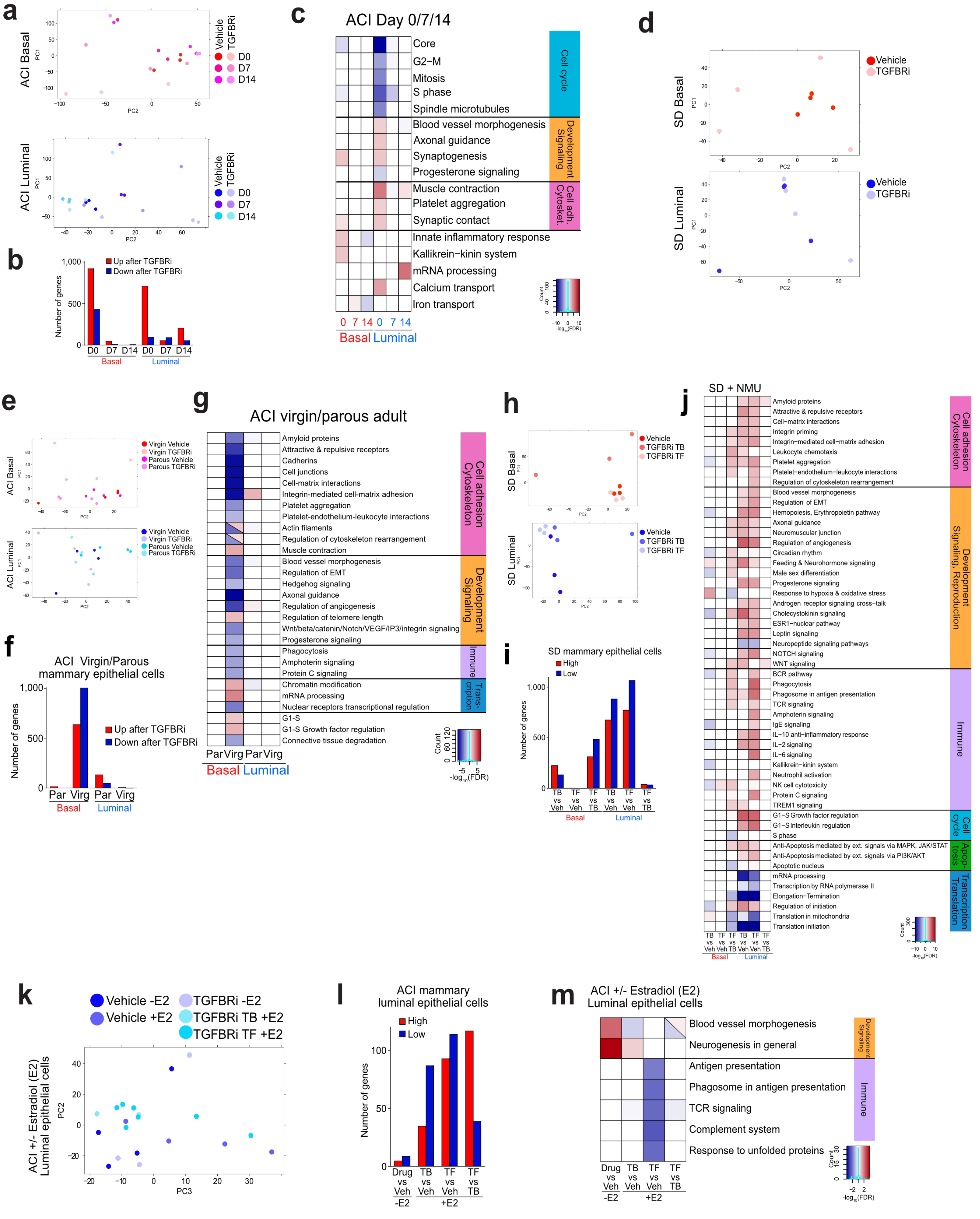
a
ACI Basal
ACI Luminal
PC1
PC2
Vehicle
TGFBRi
D0
D7
D14
b
Up after TGFBRi
Down after TGFBRi
Number of genes
1,000
500
0
D0 D7 D14 D0 D7 D14
Basal
Luminal
c
ACI Day 0/7/14
Core
G2−M
Mitosis
S phase
Spindle microtubules
Cell cycle
Blood vessel morphogenesis
Axonal guidance
Synaptogenesis
Progesterone signaling
Development Signaling
Muscle contraction
Platelet aggregation
Synaptic contact
Cell adh. Cytosket.
Innate inflammatory response
Kallikrein−kinin system
mRNA processing
Calcium transport
Iron transport
0 7 14 0 7 14
Basal Luminal
Count
-log10(FDR)
d
SD Basal
SD Luminal
Vehicle
TGFBRi
PC1
PC2
e
ACI Basal
ACI Luminal
Virgin Vehicle
Virgin TGFBRi
Parous Vehicle
Parous TGFBRi
f
ACI Virgin/Parous mammary epithelial cells
Up after TGFBRi
Down after TGFBRi
Number of genes
Par Virg Par Virg
Basal Luminal
g
ACI virgin/parous adult
Amyloid proteins
Attractive & repulsive receptors
Cadherins
Cell junctions
Cell-matrix interactions
Integrin-mediated cell-matrix adhesion
Platelet aggregation
Platelet-endothelium-leukocyte interactions
Actin filaments
Regulation of cytoskeleton rearrangement
Muscle contraction
Cell adhesion Cytoskeleton
Blood vessel morphogenesis
Regulation of EMT
Hedgehog signaling
Axonal guidance
Regulation of angiogenesis
Regulation of telomere length
Wnt/beta/catenin/Notch/VEGF/IP3/integrin signaling
Progesterone signaling
Development Signaling
Phagocytosis
Amphoterin signaling
Protein C signaling
Immune
Chromatin modification
mRNA processing
Nuclear receptors transcriptional regulation
Trans-cription
G1-S
G1-S Growth factor regulation
Connective tissue degradation
Par Virg Par Virg
Basal Luminal
Count
-log10(FDR)
h
SD Basal
SD Luminal
Vehicle
TGFBRi TB
TGFBRi TF
i
SD mammary epithelial cells
High
Low
Number of genes
TB vs Veh
TF vs Veh
TF vs TB
Basal
Luminal
j
SD + NMU
Amyloid proteins
Attractive & repulsive receptors
Cell−matrix interactions
Integrin priming
Integrin−mediated cell−matrix adhesion
Leukocyte chemotaxis
Platelet aggregation
Platelet−endothelium−leukocyte interactions
Regulation of cytoskeleton rearrangement
Cell adhesion Cytoskeleton
Blood vessel morphogenesis
Regulation of EMT
Hemopoiesis, Erythropoietin pathway
Axonal guidance
Neuromuscular junction
Regulation of angiogenesis
Circadian rhythm
Feeding & Neurohormone signaling
Male sex differentiation
Progesterone signaling
Response to hypoxia & oxidative stress
Androgen receptor signaling cross−talk
Cholecystokinin signaling
ESR1−nuclear pathway
Leptin signaling
Neuropeptide signaling pathways
NOTCH signaling
WNT signaling
Development Signaling, Reproduction
BCR pathway
Phagocytosis
Phagosome in antigen presentation
TCR signaling
Amphoterin signaling
IgE signaling
IL−10 anti−inflammatory response
IL−2 signaling
IL−6 signaling
Kallikrein−kinin system
Neutrophil activation
NK cell cytotoxicity
Protein C signaling
TREM1 signaling
Immune
G1−S Growth factor regulation
G1−S Interleukin regulation
S phase
Cell cycle
Anti−Apoptosis mediated by ext. signals via MAPK, JAK/STAT
Anti−Apoptosis mediated by ext. signals via PI3K/AKT
Apoptotic nucleus
Apop-tosis
mRNA processing
Transcription by RNA polymerase II
Elongation−Termination
Regulation of initiation
Translation in mitochondria
Translation initiation
Transcription Translation
TB vs Veh
TF vs Veh
TF vs TB
Basal
Luminal
Count
-log10(FDR)
k
Vehicle -E2
Vehicle +E2
TGFBRi -E2
TGFBRi TB +E2
TGFBRi TF +E2
ACI +/- Estradiol (E2) Luminal epithelial cells
PC2
PC3
l
ACI mammary luminal epithelial cells
High
Low
Number of genes
Drug vs Veh
TB vs Veh
TF vs Veh
TF vs TB
-E2
+E2
m
ACI +/- Estradiol (E2) Luminal epithelial cells
Blood vessel morphogenesis
Neurogenesis in general
Development Signaling
Antigen presentation
Phagosome in antigen presentation
TCR signaling
Complement system
Response to unfolded proteins
Immune
Drug vs Veh
TB vs Veh
TF vs Veh
TF vs TB
-E2
+E2
Count
-log10(FDR)

Suppl Fig 8

Supplementary Figure 8. Bulk RNA-seq of basal and luminal mammary epithelial cells after TGFBRi treatment. a-c, PCA plots of basal and luminal samples from ACI rats (**a**), bar plot of differentially expressed genes ($p_{adj} < 0.05$) (**b**), and heatmap of MetaCore networks significantly enriched (FDR < 0.1, $p < 0.001$) among upregulated and downregulated genes from ACI basal and luminal mammary epithelial samples at 0, 7, and 14 days after stopping a 10-day treatment with TGFBRi (**c**)**. d**, PCA plots of basal and luminal samples from SD rats. **e-g**, PCA plots of basal and luminal samples from adult virgin and parous ACI rats (**e**), bar plot of differentially expressed genes ($p_{adj} < 0.05$) (**f**), and heatmap of MetaCore networks significantly enriched (FDR < 0.1, $p < 0.001$) among upregulated and downregulated genes from basal and luminal mammary epithelial samples of adult virgin and parous ACI rats (**g**). Virg = virgin, Par = Parous. **h-j**, PCA plots of basal and luminal samples from SD rats treated with TGFBRi or vehicle followed by a single NMU injection (**h**), bar plot of differentially expressed genes ($p_{adj} < 0.05$) (**i**), and heatmap of MetaCore networks significantly enriched (FDR < 0.1, $p < 0.001$) among upregulated and downregulated genes from basal and luminal samples of SD rats treated with TGFBRi or vehicle followed by a single NMU injection (**j**). Samples were collected 87 days after NMU treatment. **k-m**, PCA plot of luminal samples of ACI rats treated with TGFBRi or vehicle followed by implantation of estradiol (E2)-containing (+E2) or empty (-E2) slow-release pellets (**k**), bar plot of differentially expressed genes ($p_{adj} < 0.05$) (**l**), and heatmap of MetaCore networks significantly enriched (FDR < 0.1, $p < 0.001$) among upregulated and downregulated genes (**m**). Samples were isolated 195 days after pellet implantation. TB = tumor-bearing, TF = tumor-free. Fore Metacore analyses (**c,g,j,m**) p-values are calculated hypergeometric test.

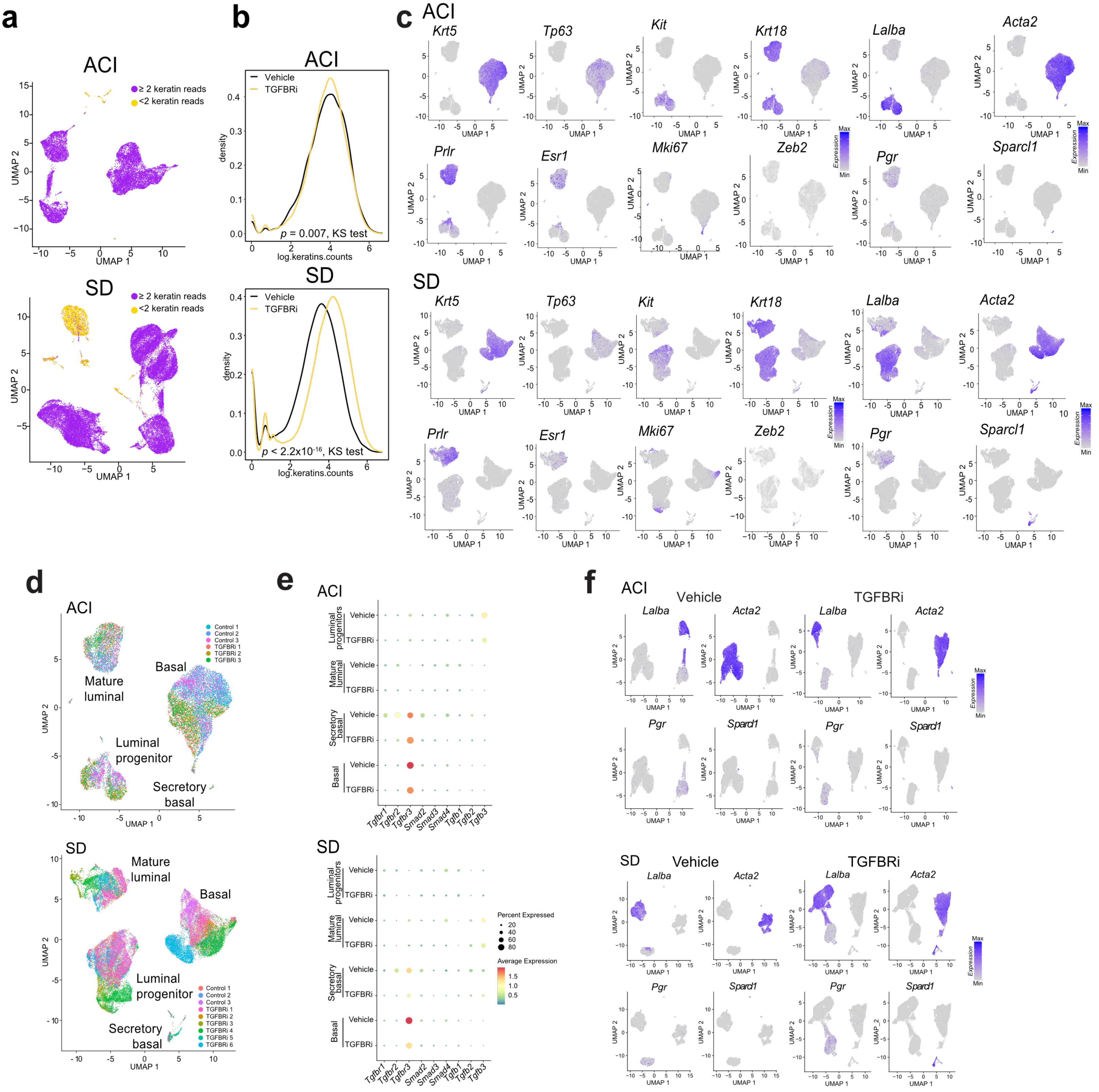
a
ACI
≥ 2 keratin reads
<2 keratin reads
UMAP 1
UMAP 2
SD
b
ACI
Vehicle
TGFBRi
density
log.keratins.counts
p = 0.007, KS test
SD
p < 2.2x10-16, KS test
c
ACI
Krt5
Tp63
Kit
Krt18
Lalba
Acta2
Prlr
Esr1
Mki67
Zeb2
Pgr
Sparcl1
Expression
Max
Min
SD
d
Control 1
Control 2
Control 3
TGFBRi 1
TGFBRi 2
TGFBRi 3
Mature luminal
Basal
Luminal progenitor
Secretory basal
TGFBRi 4
TGFBRi 5
TGFBRi 6
e
Luminal progenitors
Mature luminal
Secretory basal
Vehicle
TGFBRi
Tgfbr1
Tgfbr2
Tgfbr3
Smad2
Smad3
Smad4
Tgfb1
Tgfb2
Tgfb3
Percent Expressed
20
40
60
80
Average Expression
1.5
1.0
0.5
f
Sparcl1

Suppl Fig 9

Supplementary Figure 9. Analyses of sorted epithelial scRNA-seq data. a, UMAP plots depicting the filtering of cells based on keratin expression in ACI and SD rats. **b,** Density plots illustrating the number of reads aligned to any keratin in vehicle and TGFBRi-treated rats. P-values were calculated using Kolmogorov–Smirnov test (K-S test). **c,** UMAP plots of sorted basal and luminal cell fractions from ACI and SD rats. Cells are colored by the normalized log-transformed expression of the indicated markers. **d,** UMAP plots of sorted basal and luminal cell fractions from ACI (3 vehicle control and 3 TGFBRi-treated) and SD (3 vehicle control and 6 TGFBRi-treated) rats. Cells are colored by animal. **e**, Dot plots showing expression of genes encoding key mediators of TGFβ signaling in the four identified cell types in ACI and SD rats. **f,** UMAP plots of sorted basal and luminal cell fractions from ACI and SD rats, in data separated by treatment condition and integrated by animal. Cells are colored by the normalized log-transformed expression of the indicated markers.

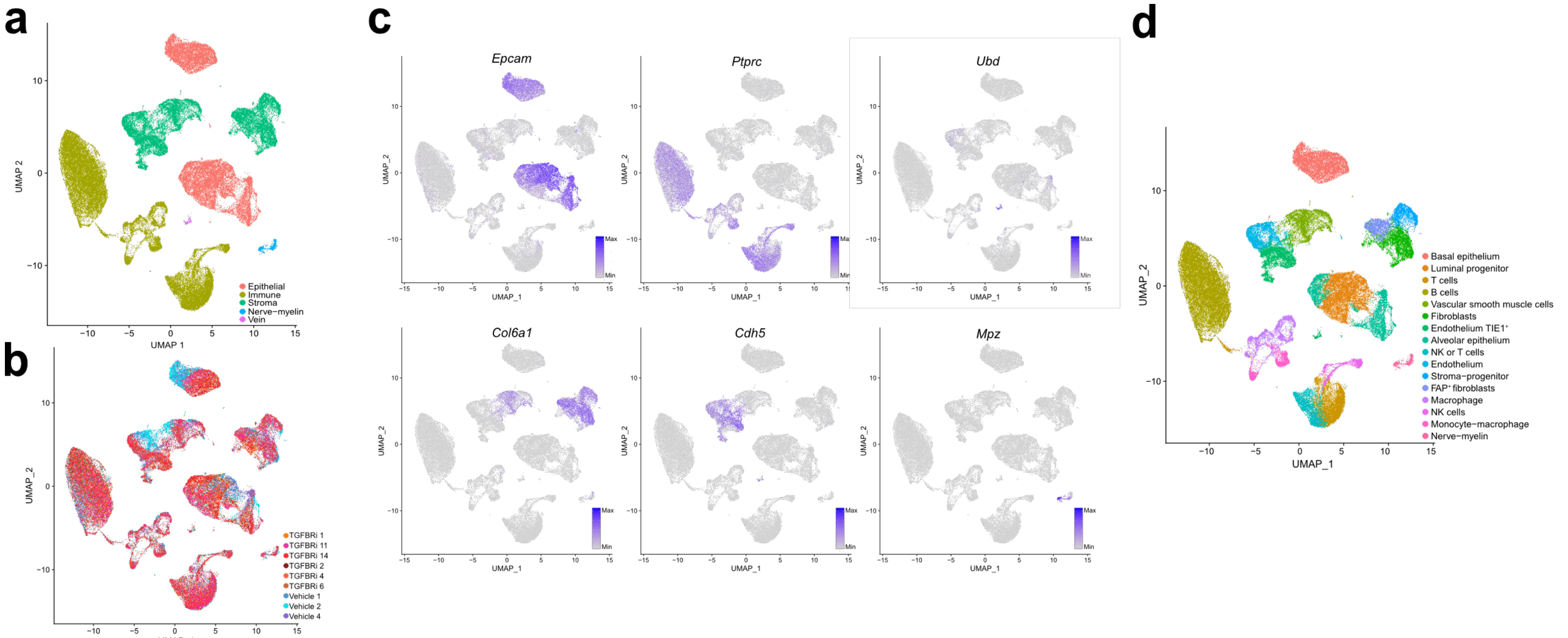

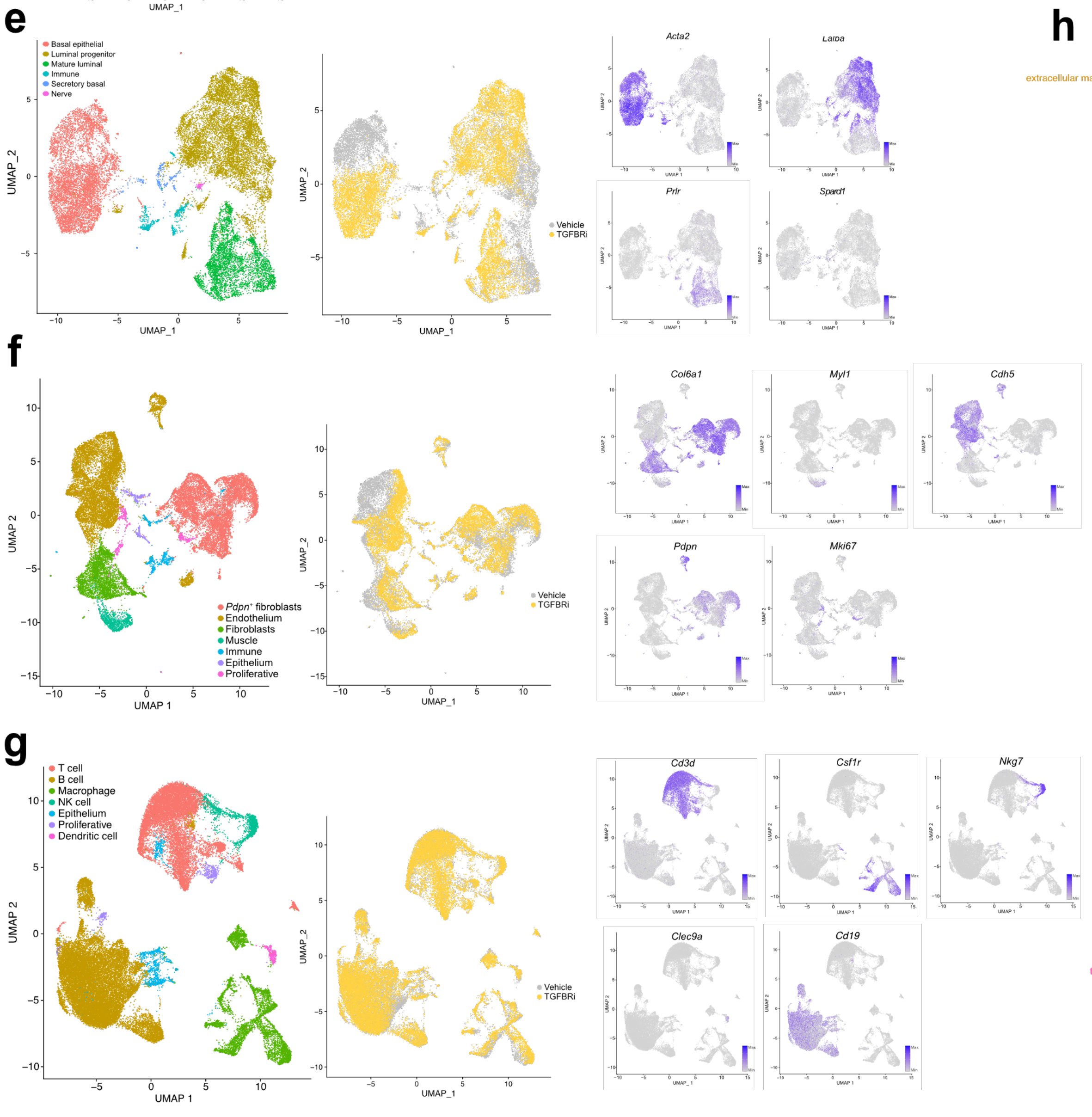

h

Basal epithelial

	pvalue	p.adjust
dendritic cell differentiation	0.0055788	0.0055788
extracellular matrix structural constituent conferring compression resistance	0.0141304	0.0141304
glomerulus vasculature development	0.0198288	0.0198288
interleukin−1 beta production	0.0107472	0.0107472
mesenchyme migration	0.0004348	0.0004348
negative regulation of muscle hypertrophy	0.0334728	0.0334728
negative regulation of tumor necrosis factor production	0.0181956	0.0181956
regulation of collagen metabolic process	0.0036058	0.0036058
skeletal muscle cell differentiation	0.0002480	0.0002480
striated muscle cell development	0.0429207	0.0429207

Luminal progenitor

	pvalue	p.adjust
brown fat cell differentiation	0.016989	0.016989
cysteine−type endopeptidase inhibitor activity	0.015427	0.015427
lactation	0.007407	0.007407
mammary gland development	0.008922	0.008922
positive regulation of insulin secretion	0.032365	0.032365
regulation of biomineral tissue development	0.045045	0.045045
regulation of biomineralization	0.045045	0.045045
regulation of smooth muscle cell differentiation	0.029462	0.029462
**SMAD protein signal transduction**	0.041714	0.041714
smooth muscle cell differentiation	0.029462	0.029462

Mature luminal

	pvalue	p.adjust
bone remodeling	0.007993	0.007993
defense response to bacterium	0.039811	0.039811
fatty acid synthase activity	0.019538	0.019538
lactation	0.000768	0.000768
lipid biosynthetic process	0.025964	0.025964
mammary gland development	0.005836	0.005836
negative regulation of MAP kinase activity	0.038885	0.038885
regulation of tissue remodeling	0.009547	0.009547
response to mineralocorticoid	0.045021	0.045021
tissue remodeling	0.009217	0.009217

Endothelium

	pvalue	p.adjust
cortical actin cytoskeleton organization	0.0319508	0.0319508
negative regulation of innate immune response	0.0257988	0.0257988
positive regulation of lymphocyte migration	0.0095257	0.0095257
**R−SMAD binding**	0.0158762	0.0158762
regulation of cellular extravasation	0.0001947	0.0001947
regulation of leukocyte adhesion to vascular endothelial cell	0.0005842	0.0005842
regulation of leukocyte tethering or rolling	0.0001973	0.0001973
regulation of T cell migration	0.0134947	0.0134947
**SMAD binding**	0.0383073	0.0383073
ventricular cardiac muscle tissue development	0.0173502	0.0173502

Fibroblasts

	pvalue	p.adjust
insulin−like growth factor II binding	0.022102	0.022102
negative regulation of MAP kinase activity	0.025518	0.025518
negative regulation of mitotic cell cycle	0.029552	0.029552
negative regulation of protein serine/threonine kinase activity	0.023363	0.023363
osteoclast differentiation	0.027375	0.027375
**R−SMAD binding**	0.005421	0.005421
response to corticosterone	0.013310	0.013310
skeletal muscle cell differentiation	0.004371	0.004371
**SMAD binding**	0.031625	0.031625
**transforming growth factor beta receptor signaling pathway**	0.037323	0.037323

Macrophage

	pvalue	p.adjust
fat cell differentiation	0.031843	0.031843
I−kappaB/NF−kappaB complex	0.031475	0.031475
interleukin−1−mediated signaling pathway	0.029291	0.029291
positive regulation of fibroblast proliferation	0.001360	0.001360
positive regulation of lymphocyte proliferation	0.044193	0.044193
regulation of insulin secretion	0.008983	0.008983
regulation of natural killer cell activation	0.030658	0.030658
regulation of vascular endothelial growth factor receptor signaling pathway	0.043803	0.043803
skeletal muscle cell differentiation	0.039774	0.039774

Suppl Fig 10

Supplementary Figure 10. Whole mammary gland scRNA-seq data. a-d, UMAP plots depicting clustering of all cells colored based on the assigned major cell types (**a**), normalized expression of relevant cell-type specific markers (**c**), animal (**b**), and detailed assigned cell types (**d**). **e**-**g,** UMAP plots depicting separate analyses of the epithelial cells (**e**), stromal (**f**), and immune cells (**g**), colored by assigned major cell subtypes, treatment, and the normalized expression of known cell subtype-specific markers.

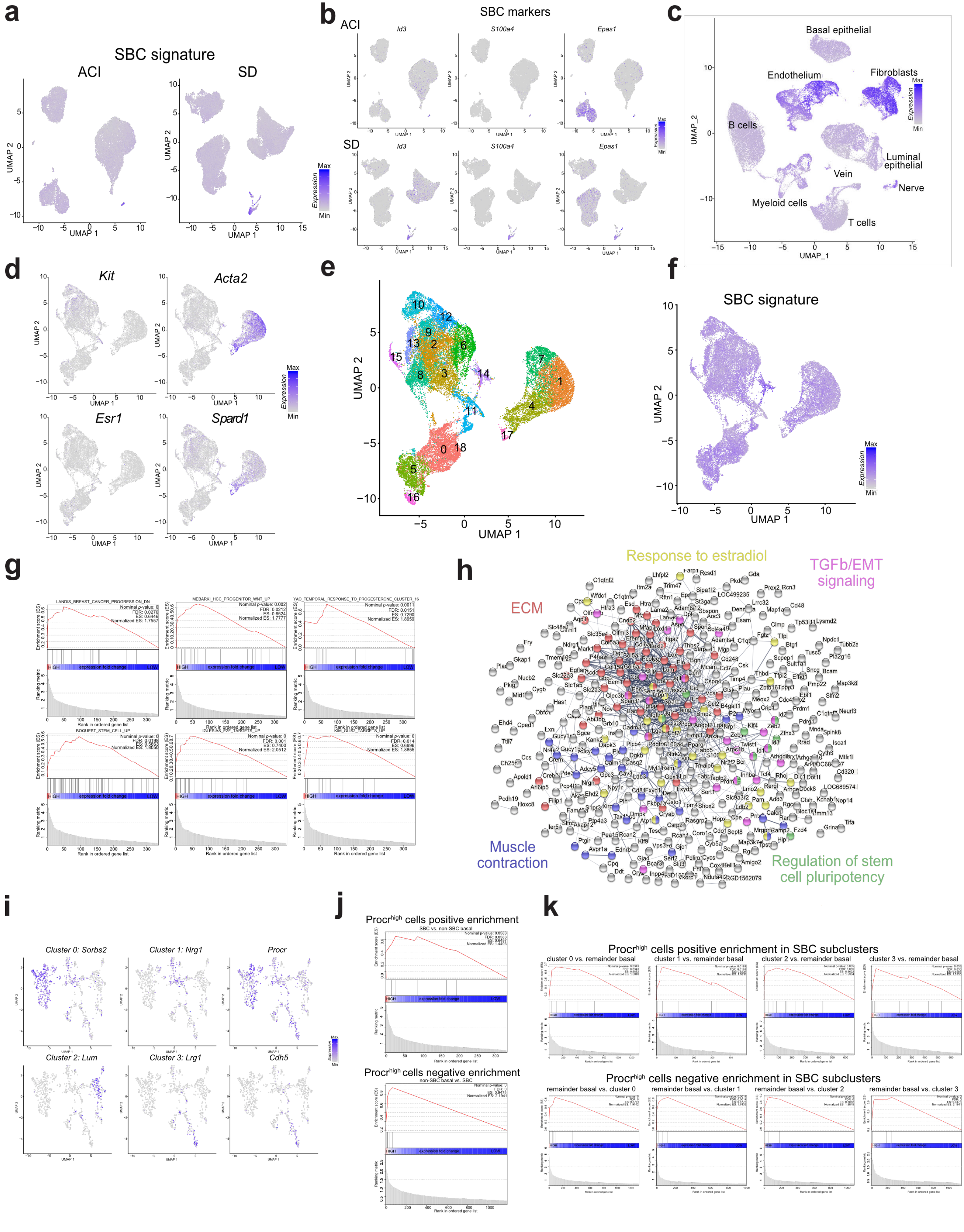
a
SBC signature
ACI
SD
UMAP 1
UMAP 2
Expression
Max
Min
b
SBC markers
ACI
SD
Id3
S100a4
Epas1
c
Basal epithelial
Endothelium
Fibroblasts
B cells
Luminal epithelial
Vein
Nerve
Myeloid cells
T cells
UMAP_1
UMAP_2
d
Kit
Acta2
Esr1
Spard1
e
f
SBC signature
g
LANDIS_BREAST_CANCER_PROGRESSION_DN
MEBARKI_HCC_PROGENITOR_WNT_UP
YAO_TEMPORAL_RESPONSE_TO_PROGESTERONE_CLUSTER_16
BOQUEST_STEM_CELL_UP
IGLESIAS_E2F_TARGETS_UP
KIM_GLIS2_TARGETS_UP
Rank in ordered gene list
Ranking metric
Enrichment score (ES)
h
Response to estradiol
TGFb/EMT signaling
ECM
Muscle contraction
Regulation of stem cell pluripotency
i
Cluster 0: Sorbs2
Cluster 1: Nrg1
Procr
Cluster 2: Lum
Cluster 3: Lrg1
Cdh5
j
Procrhigh cells positive enrichment
SBC vs. non-SBC basal
Procrhigh cells negative enrichment
non-SBC basal vs. SBC
k
Procrhigh cells positive enrichment in SBC subclusters
cluster 0 vs. remainder basal
cluster 1 vs. remainder basal
cluster 2 vs. remainder basal
cluster 3 vs. remainder basal
Procrhigh cells negative enrichment in SBC subclusters
remainder basal vs. cluster 0
remainder basal vs. cluster 1
remainder basal vs. cluster 2
remainder basal vs. cluster 3

Suppl Fig 11

Supplementary Figure 11. Functional relevance of secretory basal cells. a,b, UMAP plots of mammary epithelial cells from ACI and SD rats colored by the expression of the SBC gene signature (**a**) or by the expression of a subset of SBC markers (**b**). **c**, UMAP plot of whole mammary gland scRNA-seq data colored by the expression of the SBC gene signature. **d-f**, UMAP plot of human breast tissue scRNA-seq data[31] colored by the expression of known cell type-specific markers (**d**), assigned clusters (**e**) and SBC gene signature (**f**). **g**, GSEA enrichment plots of the SBC signature in the indicated pathways. **h**, STRING protein interaction analyses on genes specifically expressed in SBCs. Proteins with similar molecular function are color coded. **i,** UMAP plots of SBC subclusters in SD rats, colored by the expression of selected SBC subcluster-specific genes. **j,k** GSEA plots showing the enrichment of the SBC signature (**j**) or of DEGs characterizing SBC subclusters (**k**) in $Procr^{+}$ and and $Procr^{neg}$ cells from mouse mammary gland[33]. For GSEA (**g,j,k**) p-values are calculated using Kolmogorov-Smirnov test following Benjamini-Hochberg adjustment.

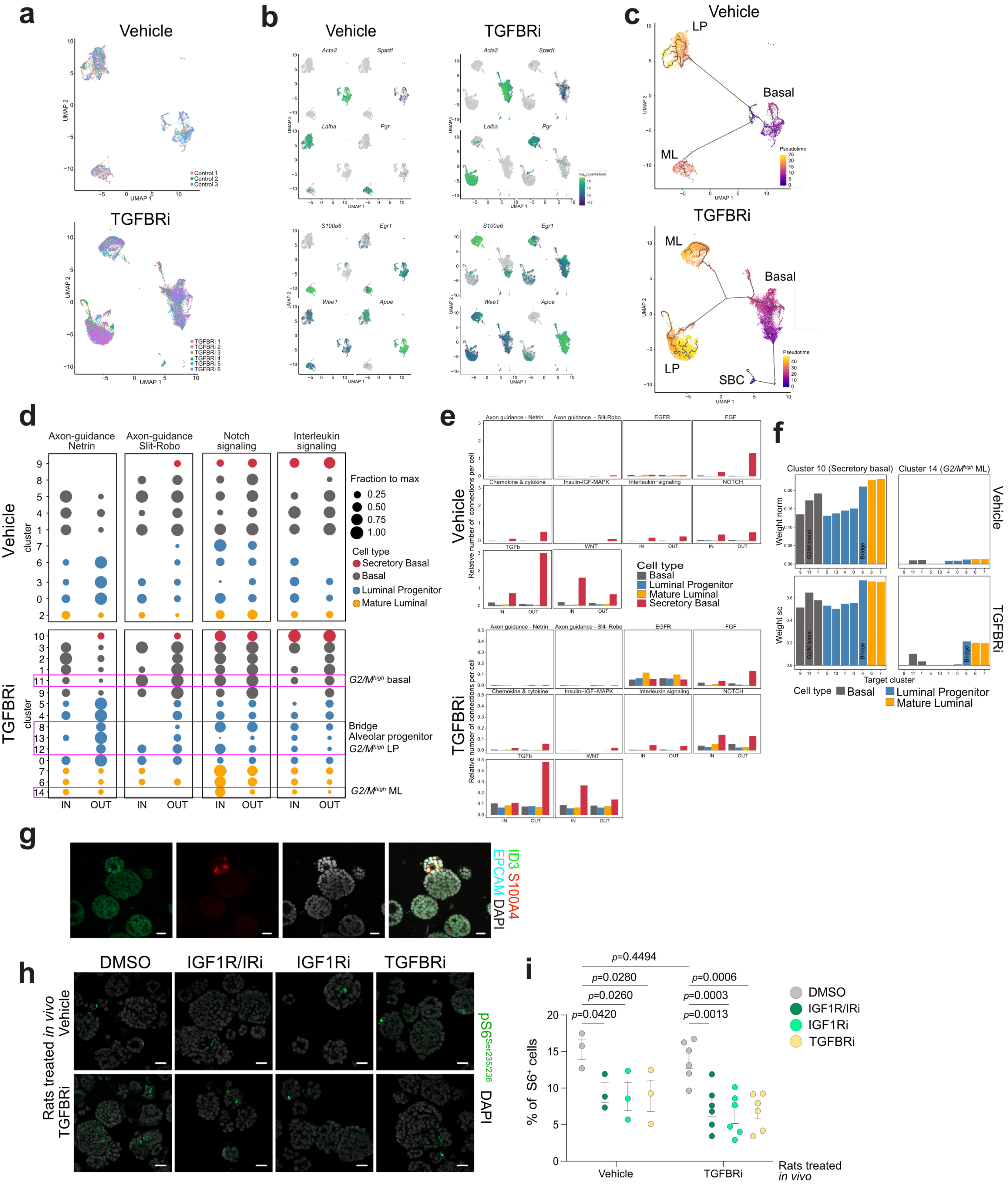

a
Vehicle
TGFBRi
b
Vehicle
TGFBRi
c
Vehicle
LP
Basal
ML
TGFBRi
ML
Basal
LP
SBC
d
Axon-guidance Netrin
Axon-guidance Slit-Robo
Notch signaling
Interleukin signaling
Fraction to max
Cell type
Secretory Basal
Basal
Luminal Progenitor
Mature Luminal
G2/M high basal
Bridge
Alveolar progenitor
G2/M high LP
G2/M high ML
IN
OUT
e
Cell type
Basal
Luminal Progenitor
Mature Luminal
Secretory Basal
f
Cluster 10 (Secretory basal)
Cluster 14 (G2/M high ML)
Target cluster
g
ID3 S100A4 EPCAM DAPI
h
DMSO
IGF1R/IRi
IGF1Ri
TGFBRi
Rats treated in vivo
Vehicle
TGFBRi
pS6 Ser235/236
DAPI
i
DMSO
IGF1R/IRi
IGF1Ri
TGFBRi
% of S6+ cells
Vehicle
TGFBRi
Rats treated in vivo

Suppl Fig 12

Supplementary Figure 12. Functional features of secretory basal cells. a,b, UMAP plots colored by animal (**a**) or by the expression of cell-type-specific markers based on which monocle3 pseudotime trajectories were inferred (**b**). **c**, Differentiation trajectory of mammary epithelial cells in control and TGFBRi-treated SD rats constructed by monocle3. Lines connecting subclusters represent the structure of the inferred Minimum Spanning Tree. The markers in (b) were used to assign cell types. **d**, Cell type-specific inferred connectivity among selected signaling pathways from interactome analysis in SD control and TGFBRi-treated rats. Single cell clusters refer to the integrated data in Figure 3c, with selected clusters highlighted. Size of dots is the fractional number of either IN or OUT connections per cluster per pathway, normalized across clusters, per pathway. **e**, Relative number of inferred interactome connections per cell and per pathway, aggregated by cell type. IN – incoming (target) and OUT – outgoing (source) signals. **f**, Average interaction intensity of the insulin-IGF-MAPK signaling pathway for the two source clusters SCBs (10) and G2/M^{high} ML (14), aggregated per target cluster. The intensity is computed as the mean of each of the two-intensity metrics returned by the tool *connectome*: weight_norm and weight_sc. **g**, Representative immunofluorescence images for ID3, S100A4, and EPCAM in rat organoids. Scale bar 20μm. n=1. The immunofluorescence was performed once, but many individual organoids were analyzed. **h,i** Representative immunofluorescence images (**h**) and quantification (**i**) of pS6 expression in rat organoids following treatment with the indicated inhibitors. Scale bar 20μm. *p*-values were calculated by two-way ANOVA (**i**). Organoids were derived from animals treated with vehicle (n=3) or TGFBRi (n=6), then treated with the indicated inhibitors in vitro. Vehicle (DMSO n=3, IGF1R/IRi n=3, IGF1Ri n=3, TGFBRi n=3), TGFBRi (DMSO n=6, IGF1R/IRi n=6, IGF1Ri n=6, TGFBRi n=6). Two wells/animal/treatment were used for quantification. Graph (**i**) is presented as mean ± s.e.m. *P*-values were calculated by two-way ANOVA. Source data are provided as a Source Data file.

Supplementary Table 1

Antibodies for immunofluorescence

Marker	Host	Isotype	Clone	Dilution	antigen retrieval pH	Vendor	Cat No.
SMA	mouse	IgG2a	1A4	1:200	9	ThermoFisher Scientific	MA1-06110
Ki67	rabbit	IgG	SP6	1:100	9	Abcam	ab16667
pHH3	rabbit	IgG	polyclonal	1:200	9	Abcam	ab5176
CCas3	rabbit	IgG	polyclonal	1:200	9	Abcam	ab49822
EpCAM	rabbit	IgG	polyclonal	1:100	9	Abcam	ab71916
ER	mouse	IgG1	6F11	1:50	9	ThermoFisher Scientific	MA5-13304
PR	rabbit	IgG	polyclonal	1:500	9	Abcam	ab16661
ID3	rabbit	IgG	polyclonal	1:1000	9	Abcam	ab236505
S100A4	rabbit	IgG	polyclonal	1:100	9	Biolegend	PRB-497P
EPAS1	mouse	IgG1	ep190b	1:100	9	LS Bio	LS-B501-100
RAM milk proteins	rabbit	IgG	polyclonal	1:1000	9	Nodric MUbio	RAM/MSP
CD163	rabbit	IgG	polyclonal	1:100	9	Abcam	ab182422
$pSMAD3^{Ser423/425}$	rabbit	IgG	polyclonal	1:100	9	LS Bio	LS-B64-50
KRT17	rabbit	IgG	polyclonal	1:200	9	Abcam	ab53707
$pS6^{Ser235/256}$	rabbit	IgG	D57.2.2E	1:100	9	Cell Signaling Technology	4858S

Antibodies for FACS

Marker	Host	Isotype	Clone	Dilution	Fluorophore	Vendor	Cat No.
CD45	mouse	IgG1	OX-1	1:100	V450	BD Biosciences	561587
CD31	mouse	IgG1	TLD-3A12	1:50	AlexaFluor647	Bio-rad	MCA1334A647
EpCAM	rabbit	IgG	polyclonal	1:100	Uncojugated	Abcam	ab71916
CD24	mouse	IgG2a	ML5	1:100	PE	Biolegend	311105
CD29	hamste	IgG	HMβ1-1	1:100	PE/Cy7	Biolegend	102221
CD45	mouse	IgG1	OX-1	1:100	PE/Cy7	Biolegend	202213
TCR α/β	mouse	IgG1	R73	1:50	FITC	Biolegend	201105
CD3	mouse	IgM	1F4	1:200	FITC	Biolegend	201403
CD11b/c	mouse	IgG2a	OX-42	1:200	FITC	Biolegend	201805
CD45RA	mouse	IgG1	OX-33	1:200	FITC	Biolegend	202305
CD90	mouse	IgG1	OX-7	1:100	PE	Biologened	202524
CD106	mouse	IgG1	MR106	1:100	PE	BD Biosciences	559229
CD34	rabbit	IgG	EP373Y	1:200	Uncojugated	Abcam	ab81289
