# Supplementary material for: Breast cancer prevention by short-term inhibition of TGFβ signaling
Source: Nat Commun. 2022 Dec 7;13:7558. doi: 10.1038/s41467-022-35043-5 (PMC9729304; doi:10.1038/s41467-022-35043-5)
Supplement: Supplementary file 2 — Reporting Summary [file 41467_2022_35043_MOESM2_ESM.pdf]

nature research

Corresponding author(s): Kornelia Polyak, Franziska Michor

Last updated by author(s): Nov 4, 2022

Reporting Summary

Nature Research wishes to improve the reproducibility of the work that we publish. This form provides structure for consistency and transparency in reporting. For further information on Nature Research policies, see our Editorial Policies and the Editorial Policy Checklist.

Statistics

For all statistical analyses, confirm that the following items are present in the figure legend, table legend, main text, or Methods section.

n/a	Confirmed	
[ ]	[x]	The exact sample size (*n*) for each experimental group/condition, given as a discrete number and unit of measurement
[ ]	[x]	A statement on whether measurements were taken from distinct samples or whether the same sample was measured repeatedly
[ ]	[x]	The statistical test(s) used AND whether they are one- or two-sided<br>*Only common tests should be described solely by name; describe more complex techniques in the Methods section.*
[ ]	[x]	A description of all covariates tested
[ ]	[x]	A description of any assumptions or corrections, such as tests of normality and adjustment for multiple comparisons
[ ]	[x]	A full description of the statistical parameters including central tendency (e.g. means) or other basic estimates (e.g. regression coefficient) AND variation (e.g. standard deviation) or associated estimates of uncertainty (e.g. confidence intervals)
[ ]	[x]	For null hypothesis testing, the test statistic (e.g. *F*, *t*, *r*) with confidence intervals, effect sizes, degrees of freedom and *P* value noted<br>*Give P values as exact values whenever suitable.*
[x]	[ ]	For Bayesian analysis, information on the choice of priors and Markov chain Monte Carlo settings
[x]	[ ]	For hierarchical and complex designs, identification of the appropriate level for tests and full reporting of outcomes
[x]	[ ]	Estimates of effect sizes (e.g. Cohen's *d*, Pearson's *r*), indicating how they were calculated

Our web collection on statistics for biologists contains articles on many of the points above.

Software and code

Policy information about availability of computer code

Data collection	For flow cytometric analysis the BD LSRFortessa (Becton Dickinson) was used. For FACS the BD FACSAria II SORP UV (Becton Dickinson) was used. Images were obtained with Pannoramic MIDI II digital slide scanner (3DHistech), Nikon Ti/E inverted microscope or dual 12MP wide-angle and telephoto camera. Absorbances for ELISAs were measured using Infinite 200Pro instrument and the software Tecan i-control 1.10.4.0. Total RNA was measured by Agilent 2100 Bioanalyzer, dsDNA concentrations were measured by Qubit Fluorometer, the size of library fragment was measured by Agilent TapeStation 2200. Bulk RNA sequencing was performed using a Illumina NextSeq500 instrument. scRNA sequencing was performed using the 10x 3' v3 (sorted cells) and 10x 5' v2 (whole mammary gland) platforms.
Data analysis	Statistical analyses were performed using GraphPad Prism (v8.1.1) or R (v3.6.2). Flow cytometry data were analyzed using FlowJo v10.6.2 (Becton Dickinson & Company). Images were analyzed by ImageJ 1.53 (Fiji) or QuPath (v0.1.2). RNAseq data were analyzed using STAR (version STAR_2.5.1b), HTSeq-count (version 0.6.1p1), DESeq2 (v1.20.0), and MetaCore (v19.4). Single cell RNA-seq data were analyzed using CellRanger (v4.0.0-sorted cells and v6.0.0-whole mammary gland) and Seurat (v2.3.4). All code used to analyze genomics data and produce the corresponding figures, as well as Zenodo links to preprocessed genomics data objects, are available on the GitHub repository https://github.com/csimona/tumor-prevention-rat-scRNAseq.

For manuscripts utilizing custom algorithms or software that are central to the research but not yet described in published literature, software must be made available to editors and reviewers. We strongly encourage code deposition in a community repository (e.g. GitHub). See the Nature Research guidelines for submitting code & software for further information.

Data

Policy information about availability of data

All manuscripts must include a data availability statement. This statement should provide the following information, where applicable:

- Accession codes, unique identifiers, or web links for publicly available datasets
- A list of figures that have associated raw data
- A description of any restrictions on data availability

The human (Pal B, et al. EMBO J 40, e107333, 2021). and mouse (Bach et al. Nature Comm 8, 2128, 2017) publicly available single cell RNA-seq data used in this study are available in the GEO database under accession numbers GSE106273 (https://www.ncbi.nlm.nih.gov/geo/query/acc.cgi?acc=GSE106273) and GSE161529 (https://www.ncbi.nlm.nih.gov/geo/query/acc.cgi?acc=GSE161529). The RNA-seq and scRNA-seq data generated in this study have been deposited in the NCBI GEO database under accession number GSE184095. The remaining data are available within the Article, Supplementary Information or Source Data file.

Field-specific reporting

Please select the one below that is the best fit for your research. If you are not sure, read the appropriate sections before making your selection.

- [x] Life sciences
- [ ] Behavioural & social sciences
- [ ] Ecological, evolutionary & environmental sciences

For a reference copy of the document with all sections, see nature.com/documents/nr-reporting-summary-flat.pdf

Life sciences study design

All studies must disclose on these points even when the disclosure is negative.

Sample size	Sample sizes were chosen based on our prior studies using the same types of assays (https://pubmed.ncbi.nlm.nih.gov/18455123/) as well as based on prior publications (https://doi.org/10.1093/ilar.43.4.207) to ensure statistically significant results.<br>For long-term animal experiments we started with sample sizes of at least 10 rats per group, and used two different rat strains. For short-term, we performed most experiments in triplicates, replicated with other cohorts, and in two different rat strains.<br>For immunofluoresece experiments, we quantified at least three random areas per animal.<br>For FACS and flow cytometric analysis, we used a minimum of three animals per group.<br>For ELISA, we tested two separate dilutions in triplicates<br>Bulk RNA-seq was performed on triplicates or quadruplicates<br>scRNA-seq for sorting cells was performed on about 5,000 cells from three individual animals (triplicates) per group<br>scRNA-seq for the whole mammary gland was performed on about 10,000 cells from three individual animals (triplicates) per group.
Data exclusions	A few ACI rats were sensored in the tumor experiment (E2-induced tumors) due to morbidity from pituitary tumors. This was noted in the survival curved and explained in the text. Aditionally, if animals died due to technical issues with gavage, they were excluded from the experiment.
Replication	As mentioned in the manuscript, we sometimes could not replicate the drop in basal or luminal fractions upon TGFBRi treatment using FACS or flow cytometric analysis. We found this method of quantification using flow cytometry to be variable, depending on length and method of mammary gland digestion and whether cells went through one freeze-thaw cycle before analysis.<br>For all other experiments, all attempts at replication were successful. We replicated all results at least once in the other rat strain, like scRNA-seq and tumor studies. These samples were performed once due to time and cost limitations. Bulk RNAseq was performed twice for both strains at time point D0. Short-term animal experiments were done in multiple independent cohorts and most subsequent analyses (IF, ELISA, FACS/flow cytometry) were performed on samples from at least two independent cohorts in both rat strains.
Randomization	Rats were randomized to treatment groups right before treatment initiation with TGFBRi to ensure that size/weight variability among animals was the same in each group.
Blinding	Histological analyses by rodent pathologist were blinded. FACS was performed by FACS facility staff who were blinded to the identity or meaning of the samples. Similarly, some RNA-seq analysis was performed by bioinformaticians blinded to the identity of the samples. For immunofluorescence, ELISA, flow cytometry studies and organoid studies, blinding was not possible, since samples had to be labeled to be able to match the data to the correct animals. However, these assays were performed by multiple independent personnel participating in the study at different times during the project with the same results.

Reporting for specific materials, systems and methods

We require information from authors about some types of materials, experimental systems and methods used in many studies. Here, indicate whether each material, system or method listed is relevant to your study. If you are not sure if a list item applies to your research, read the appropriate section before selecting a response.

Materials & experimental systems

n/a	Involved in the study	
☐	☒	Antibodies
☒	☐	Eukaryotic cell lines
☒	☐	Palaeontology and archaeology
☐	☒	Animals and other organisms
☐	☒	Human research participants
☒	☐	Clinical data
☒	☐	Dual use research of concern

Methods

n/a	Involved in the study	
☒	☐	ChIP-seq
☐	☒	Flow cytometry
☒	☐	MRI-based neuroimaging

Antibodies

Antibodies used	See Supplementary Table 1 for antibody clone and ordering information.<br>Antibodies for immunofluorecence: phospho-Histone H3 (pHH3, polyclonal, 1:200), Ki67 (clone SP6, 1:100), cleaved caspase-3 (clCASP3, polyclonal, 1:200), CD163 (polyclonal, 1:100), SMA (clone 1A40, 1:200), EPCAM (polyclonal, 1:100), ER (clone 6F11, 1:50), PR (polyclonal, 1:500), ID3 (polyclonal, 1:1000), S100A4 (polyclonal, 1:100), EPAS1 (clone ep190b, 1:100), RAM milk proteins (polyclonal, 1:1000), pSMAD3Ser423/425 (polyclonal, 1:100), KRT17 (polyclonal, 1:200), pS6Ser235/256 (clone D57.2.2E, 1:100)<br>Antibodies for FACS/flow cytometry of mammary glands: CD24 (clone ML5, 1:100), CD29 (HMβ1-1, 1:100), CD31 (clone TLD-3A12, 1:50), CD45 (clone OX-1, 1:100)<br>Antibodies for FACS of tumors: CD45 (clone OX-1, 1:100) , EPCAM (polyclonal, 1:100)<br>Antibodies for flow cytometry of bone marrow cells: CD45 (clone OX-1, 1:100), TCR α/β (R73, 1:50), CD3 (1F4, 1:200), CD11b/c (OX-42, 1:200), CD45ra (OX-33, 1:200), CD90 (OX-7, 1:100), CD106 (MR106, 1:100), CD34 (EP373Y, 1:200)
Validation	Antibodies used for FACS staining were validated on rat tissues, and the obtained percentages corresponded well to the expected values. IHC and IF antibodies were validated on appropriate FFPE rat tissue and tumors. In all cases, positive and negative controls were used with tissues expected to express or not express the markers or interest, respectively.

Animals and other organisms

Policy information about studies involving animals; ARRIVE guidelines recommended for reporting animal research

Laboratory animals	4-week-old virgin female Sprague-Dawley (Hsd:Sprague Dawley SD) as well as 4- to 5-week-old virgin and 18- to 24-week-old virgin and parous female ACI (ACI/SegHsd) female rats were purchased from Envigo.
Wild animals	No wild animals were used in the study.
Field-collected samples	No field collected samples were used in the study.
Ethics oversight	All animal experiments were performed following protocol #15-050 approved by the DFCI Institutional Animal Care & Use Committee (IACUC).

Note that full information on the approval of the study protocol must also be provided in the manuscript.

Human research participants

Policy information about studies involving human research participants

Population characteristics	Normal breast tissue samples were collected from women (age >18) undergoing reduction mammoplasty or prophylactic mastectomy at Dana-Farber Cancer Institute/Birgham and Women's Hospital.
Recruitment	Patients were not specifically recruited for this study, they were receiving standard of care at Dana-Farber Cancer Institute/ Birgham and Women's Hospital.
Ethics oversight	All clinical samples and data were collected following approval by Dana-Farber Cancer Institute (DFCI; IRB, protocols 08-010, 10-458, 93-085, 14-400), Institutional Review Board.

Note that full information on the approval of the study protocol must also be provided in the manuscript.

Flow Cytometry

Plots

Confirm that:

- [x] The axis labels state the marker and fluorochrome used (e.g. CD4-FITC).
- [x] The axis scales are clearly visible. Include numbers along axes only for bottom left plot of group (a 'group' is an analysis of identical markers).
- [x] All plots are contour plots with outliers or pseudocolor plots.
- [x] A numerical value for number of cells or percentage (with statistics) is provided.

Methodology

Sample preparation	Mammary glands (inguinal/abdominal + thoracic) were harvested from freshly sacrificed rats. The tissue was immediately digested in a collagenase solution (2mg/ml collagenase in DMEM/F12 from Gibco/ThermoFisher) with constant stirring at 37C for 1-2 hours followed by subsequent analyses or freezing viably in 10% DMSO/FBS. The day of analysis, samples were thawed, trypsinized to obtain single cells, and stained with the appropriate antibodies. Bone marrow was collected (by flushing the femurs) and frozen viably in 10% DMSO/FBS for subsequent flow cytometry analysis. For the tumor prevention experiments, tumors were also excised. A small slice of each tumor was saved for paraffin embedding, while the rest of the tumor was digested and frozen viably for FACS. If the tumor was too small for both procedures, then it was only fixed for histological analysis.
Instrument	For flow cytometric analysis the BD LSRFortessa (Becton Dickinson) was used. For FACS the BD FACSAria II SORP UV (Becton Dickinson) was used
Software	BD FACSDiva software was used for acquiring all the data. FlowJo v 10.6.2 (Becton Dickinson & Company) was used for analysis.
Cell population abundance	Gates were conservative and consistent across samples to ensure sample purity which was confirmed by RNA-seq. We collected at least 10,000 cells.
Gating strategy	Basal & luminal mammary epithelial cells: FSC/SSC-area, -height, and -width gates were used to select live cells and discriminate doublets. Then CD45 and CD31 were used to gate out leukocytes and endothelial cells, respectively, which formed clear, discrete cell clusters in 2-D plots. CD45-CD31- cells were then displayed in 2-D plots with CD29 and CD24 on the axes. Clear, discrete CD24+CD29- and CD24+CD29+ cell populations formed. Gating strategy is shown in Supplementary Fig. 1l.<br>Tumor cells and leukocytes: FSC/SSC-area, -height, and -width gates were used to select live cells and discriminate doublets. In addition, Aqua was used to further exclude any dead cells. Then CD45 and EPCAM were used to separate leukocytes and tumor cells, respectively. The two cell populations formed clear, discrete cell clusters in 2-D plots. Gating strategy is shown in Supplementary Fig. 6h.<br>Bone marrow cells: FSC-area vs SSC-area was used to select the viable population around the clear leukocyte cluster. CD45 and Linage-negative markers were used to select a CD45+Lin- subpoulation that formed a distinct cluster in that quadrant. Then two separate gating strategies were employed. One used separated on the basis of CD106 vs SCC-A. This yielded a cell population representing a tail from the main cluster. The other strategy used CD90 and CD34 to gate a rare CD90+CD34+ population which was also a tail from the main CD90+ cell cluster. Gating strategies are shown in Supplementary Fig. 3f and 3j.

- [x] Tick this box to confirm that a figure exemplifying the gating strategy is provided in the Supplementary Information.
