# Supplementary material for: Breast cancer prevention by short-term inhibition of TGFβ signaling
Source: Nat Commun. 2022 Dec 7;13:7558. doi: 10.1038/s41467-022-35043-5 (PMC9729304; doi:10.1038/s41467-022-35043-5)
Supplement: Supplementary file 3 — Description of Additional Supplementary Files [file 41467_2022_35043_MOESM3_ESM.pdf]

Title: Supplementary Data 1.

Description: Statistical analyses of non-mammary organs of peripubertal ACI and SD rats and CIBERSORT of mammary gland CD45+ cell bulk RNA-seq data.

Title: Supplementary Data 2.

Description: Differentially expressed genes in EPCAM+ and CD45+ cells between TGFBRi- and vehicle-treated SD rats by RNA-seq and CIBERSORT analyses.

Title: Supplementary Data 3.

Description: Differentially expressed genes in bulk RNA-seq samples of mammary epithelial cells under different experimental conditions.

Title: Supplementary Data 4.

Description: MetaCore analyses from bulk RNA-seq data.

Title: Supplementary Data 5.

Description: Differentially expressed genes characterizing clusters in the single cell data, separately for control and TGFBRi-treated cells, in both ACI and SD strains.

Title: Supplementary Data 6.

Description: Differentially expressed genes in each of the cell types strongly affected by TGFBRi treatment.

Title: Supplementary Data 7.

Description: Secretory basal cell signature and subclusters in the SD strain, and TEB and ductal gene signatures.

Title: Supplementary Data 8.

Description: Signaling pathways downloaded from PANTHER for the interactome analysis. Genes highlighted in red were manually added based on published signaling pathway maps.
